# Coordination-Enhanced Luminescence on Tetra-Phenylethylene-Based Supramolecular Assemblies

**DOI:** 10.3390/molecules23020363

**Published:** 2018-02-09

**Authors:** Qian-Qian Yan, Shao-Jun Hu, Guang-Lu Zhang, Ting Zhang, Li-Peng Zhou, Qing-Fu Sun

**Affiliations:** 1 College of Chemistry, Fuzhou University, Fuzhou 350108, China; yanqianqian@fjirsm.ac.cn (Q.-Q.Y.); sjhu@fjirsm.ac.cn (S.-J.H.); 2 State Key Laboratory of Structural Chemistry, Fujian Institute of Research on the Structure of Matter, Chinese Academy of Sciences, Fuzhou 350002, China; glzhang@sdnu.edu.cn (G.-L.Z.); zhangting@fjirsm.ac.cn (T.Z.); zhoulp@fjirsm.ac.cn (L.-P.Z.)

**Keywords:** tetraphenylethylene, coordination self-assembly, fluorescence enhancement, AIE

## Abstract

Materials with aggregation-induced emission (AIE) properties have received increased attention recently due to their potential applications in light-emitting devices, chemo/biosensors and biomedical diagnostics. In general, AIE requires the forced aggregation of the AIEgens induced by the poor solvent or close arrangement of AIEgens covalently attached to polymer chains. Here, we report two coordination-enhanced fluorescent supramolecular complexes featuring hierarchically restricted intramolecular motions via the self-assembly of tetraphenylethylene (TPE)-based tetra-dentate (**L^a^**) and bidentate (**L^b^**) ligands and the *cis*-Pd(en)(NO_3_)_2_ (en = ethylenediamine) unit. While the free ligands are non-emissive in dilute solution and show typical AIE properties in both mixed solvent system and the solid state, the self-assembled complexes maintain their fluorescent nature in the solution state. In particular, the Pd_4_(**L^a^**)_2_ complex shows remarkable 6-fold fluorescent enhancement over **L^a^** in dilute solution. We anticipate that these kinds of coordination-enhanced emissive supramolecules will find applications in biomedical sensing or labeling.

## 1. Introduction

Aggregation-caused quenching (ACQ) phenomena of luminescent materials are well known, and hinder many of their applications, such as OLED, chemo- or bio-labeling and sensing. Contrary to ACQ, aggregation-induced emission (AIE) materials, which were first introduced by Tang and coworkers in 2001 [1,2], are non-emissive in dilute solutions, but become highly luminescent in their aggregated state. Molecules with AIE properties are called AIEgens, from which a variety of functional materials, such as metal-organic frameworks (MOFs) [3,4], covalent-organic frameworks (COFs) [5], and co-polymers [6,7] have been designed. Luminescent properties of these materials have shown rich stimuli-responsiveness towards light, aggregation, chemicals, heat, mechanical forces, etc. However, most of the known AIE materials rely on the high local concentration of the AIEgens. It is thus necessary to search for efficient strategies to “turn on” the AIE at low concentrations. To achieve this, tight covalent bonding of AIE chromophores into one molecule has been shown to be an effective strategy, but this usually requires multi-step and/or low-yield covalent synthesis.

Tetraphenylethylene (TPE) is a popular AIE-active fluorophore, whose fluorescence can be induced by restricting C=C bond twist and phenyl-ring rotation [8,9]. Many strategies, including covalent modifications, host-guest chemistry, anion coordination, π-π stacking, electrostatic interactions and embedding into MOFs, have been developed to “turn on” the emission [10,11]. In particular, construction of MOFs by anchoring TPE chromophores to metal ions within a rigid matrix has been shown to be an effective alternative strategy to get strongly luminescent nanoparticles [12,13]. However, the insolubility of MOFs poses inherent problems for their fabrication.

Recently, coordination-directed self-assembly has been proven to be a powerful supramolecular approach to organize multiple AIE chromophores onto the discrete supramolecular coordination complexes (SCCs). For example, Stang and coworkers have reported a series of light-emitting tetraphenylethylene (TPE)-based supramolecular platforms and their multifaceted applications in sensor devices and advanced optoelectronic materials [14]. The Su group has reported a tetraphenylethylene (TPE)-based tetragonal Ag_4_L_2_ cage with luminescent behavior that is significantly different from that of both AIE and ACQ mechanisms [15]. Luminescent SCCs materials have many advantages compared to MOFs, such as the circumvention of synthetic steps, inherent error-correction and defect-free assembly, facile and fast formation, increased solubility and so on. In addition, the finite nature of SCC structures facilitates convenient solution-based characterization/fabrication methods.

**Scheme 1:**
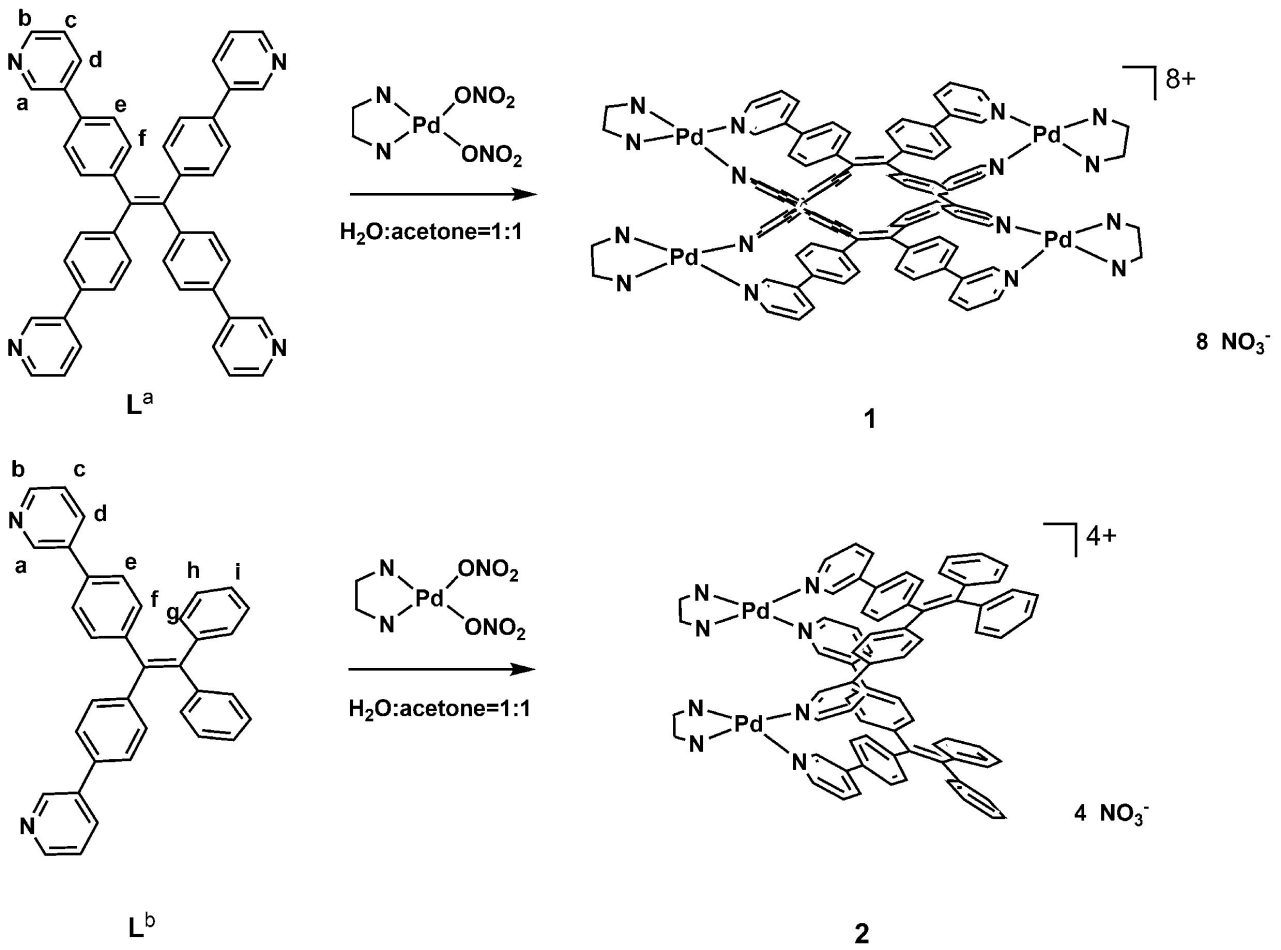
Coordination-driven self-assembly of supramolecular complexes **1** and **2** from two TPE-based ligands **L^a,b^** and the *cis*-blocked (en)Pd(NO_3_)_2_ clips.

Although several light-emitting TPE-based discrete organoplatinum metallacages and metallacycles have been reported [16,17,18,19,20], AIE-active organopalladium SCCs have rarely been explored [21,22], even though palladium has a lower cost than platinum. Pd^2+^ is a well-known fluorescence quencher due to heavy atom effects and its 4d^8^ electronic configuration, the photo induced electron transfer and photo induced energy transfer may both exist through the interactions between fluorophore and Pd^2+^ [23]. Lots of fluorescence probes for detecting Pd^2+^ based on Pd^2+^ coordination-induced fluorescence quenching have been designed [24,25]. Herein, we report the coordination-driven self-assembly and the luminescent studies of two SCCs (**1**, **2**) from the TPE-based polypyridine ligands (**L^a^** and **L^b^**) and the (en)Pd(NO_3_)_2_ (en = ethylenediamine) clipping units (Scheme 1). While the free ligands are non-emissive in dilute solution and show typical AIE properties in the aggregated states, the self-assembled complexes maintain their fluorescent nature in dilute solution. Such a coordination-enhanced emission behavior has been clearly confirmed by comparison of the two SCC_S_ featuring hierarchically restricted intramolecular motions. In particular, complex **1** shows a remarkable 6-fold fluorescent enhancement over **L^a^** in dilute solution.

## 2. Results and Discussion

**Figure 1:**
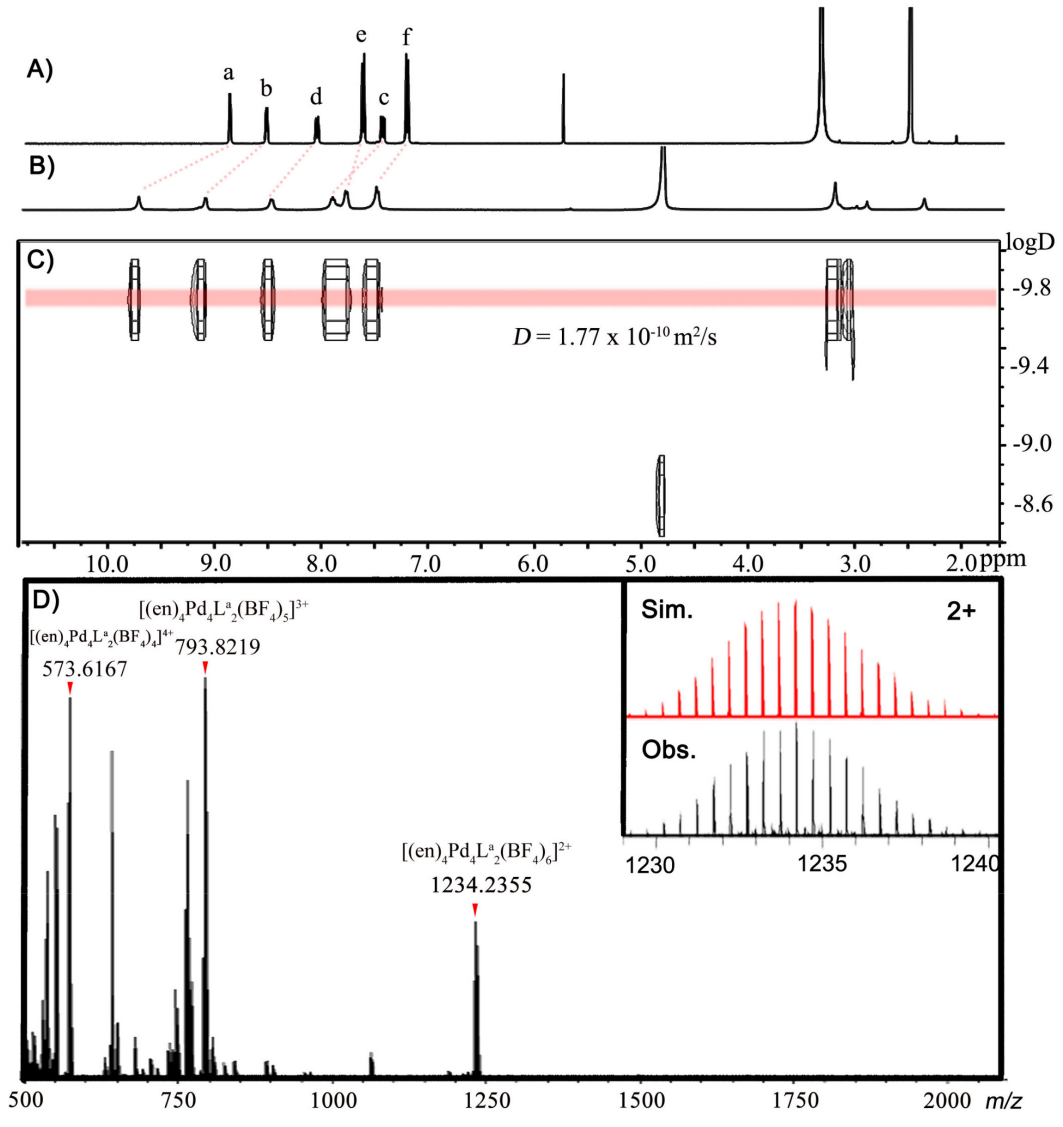
^1^H-NMR spectra of (**A**) ligand **L^a^** (400 MHz, DMSO-*d*_6_, 298 K), (**B**) complex **1** (400 MHz, D_2_O:Acetone-*d*_6_ = 1:1, 298 K); (**C**) ^1^H DOSY spectrum and (**D**) ESI-TOF-MS of complex **1** with insets showing the observed (Obs.) and simulated (Sim.) isotopic patterns of the 2+ peak.

For comparison purposes, ligands **L^a^** and **L^b^**, with four and two pyridine coordination sites, respectively, were synthesized in two steps starting from the TPE precursor by following the reported procedures [26,27,28]. Both were fully characterized by multi-dimensional NMR and high-resolution ESI-TOF-MS spectroscopy (See SI for details). Coordination complex **1·NO_3_** was obtained quantitatively by reacting ligand **L^a^** with two equiv. of *cis*-(en)Pd(NO_3_)_2_ in a H_2_O and acetone (*v*:*v* = 1:1) mixture at 50 °C for 3 h with continuous stirring. With the progression of the self-assembly, the suspension of the ligand gradually turned into a clear yellow solution. ^1^H-NMR first confirmed the quantitative formation of a highly symmetrical complex with the observation of a single set of ligand signals (Figure 1B and Figure S8), which could be fully assigned based on a ^1^H-^1^H COSY experiment (Figures S6 and S9). The diffusion-ordered spectroscopy (DOSY) NMR spectrum (Figure 1C) [29] suggests the formation of a single product with a single diffusion coefficient of 1.77 × 10^−10^ m^2^·s^−1^, with an estimated diameter of 2.1 nm for the complex in solution.

In order to facilitate mass spectra measurement, **1·NO_3_** was transformed into its tetrafluoroborate salt (**1·BF_4_**) by precipitation with excess amount of NaBF_4_ (See SI for details). The composition of complex **1·BF_4_** as (en)_4_Pd_4_**L^a^**_2_(BF_4_)_8_ was then clearly provided by electro-spray-ionization time-of-flight mass-spectroscopy (ESI-TOF-MS) measurements. The prominent peaks observed at *m*/*z* = 1234.2355, 793.8219, 573.6167, correspond to [M − (BF_4_^−^)*_n_*]*^n^*^+^ (*n* = 2–4). Moreover, the isotopic patterns of each peak were in good agreement with the calculated values, which further confirmed the molecular formula. Similarly, reacting ligand **L^b^** with an equimolar amount of *cis*-(en)Pd(NO_3_)_2_ followed by counter-ion exchange with NaBF_4_ lead to the quantitative formation of the Pd_2_**L^b^**_2_ complex (**2·BF_4_**), which was fully characterized by multi-dimensional NMR and ESI-TOF-MS (see SI for details).

**Figure 2:**
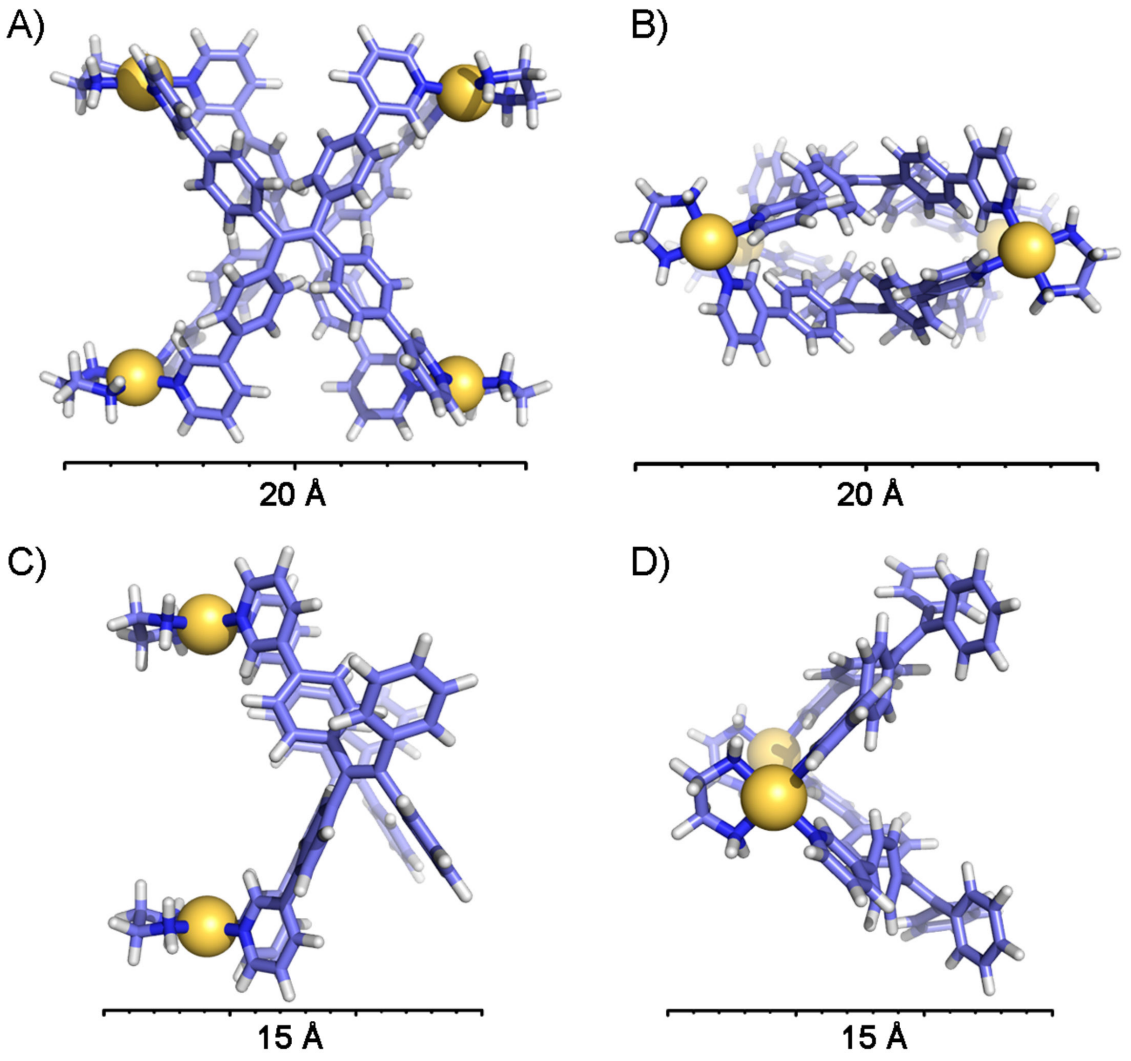
Simulated molecular structure models for complexes **1** (**A**,**B**) and **2** (**C**,**D**). Color scheme: C, slate sticks; H, white sticks; N, blue sticks; Pd, yellow spheres.

Unfortunately, efforts to grow high-quality single crystals for both compounds were failed. Therefore, molecular-mechanical simulations were performed to get an overview of the structures for complexes **1** and **2** (Figure 2). In complex **1**, as all the four pyridine arms are clipped together by the (en)Pd units, the two PTE fluorophores are forced to stack to each other, with an approximate 6.06 Å separation between the ethene groups (defined by the closest C-C distance). In contrast, the two ethene groups on the two ligands of **L^b^** in the optimized structure of complex **2** are more separated (9.51 Å apart). Such a gradual coordination-driven proximity of the TPE groups on these complexes persuaded us to investigate their differences in photoluminescent properties.

UV-Vis absorption spectra for both **L^a^** and **L^b^** (DMSO solution, 50.00 µM) were first measured, and showed broad intense band for the π-π * transitions from 270 to 400 nm (λ_max_ of **L^a^** at 286 nm, with molar absorptivity up to 4.55 × 10^4^ M^−1^⋅cm^−1^; λ_max_ of **L^b^** at 301 nm, with molar absorptivity up to 3.09 × 10^4^ M^−1^⋅cm^−1^). Significant red-shifting in the absorption spectra of complexes **1** and **2** was noticed, as a result of coordination to the (en)Pd units (Figure S15). 

**Figure 3:**
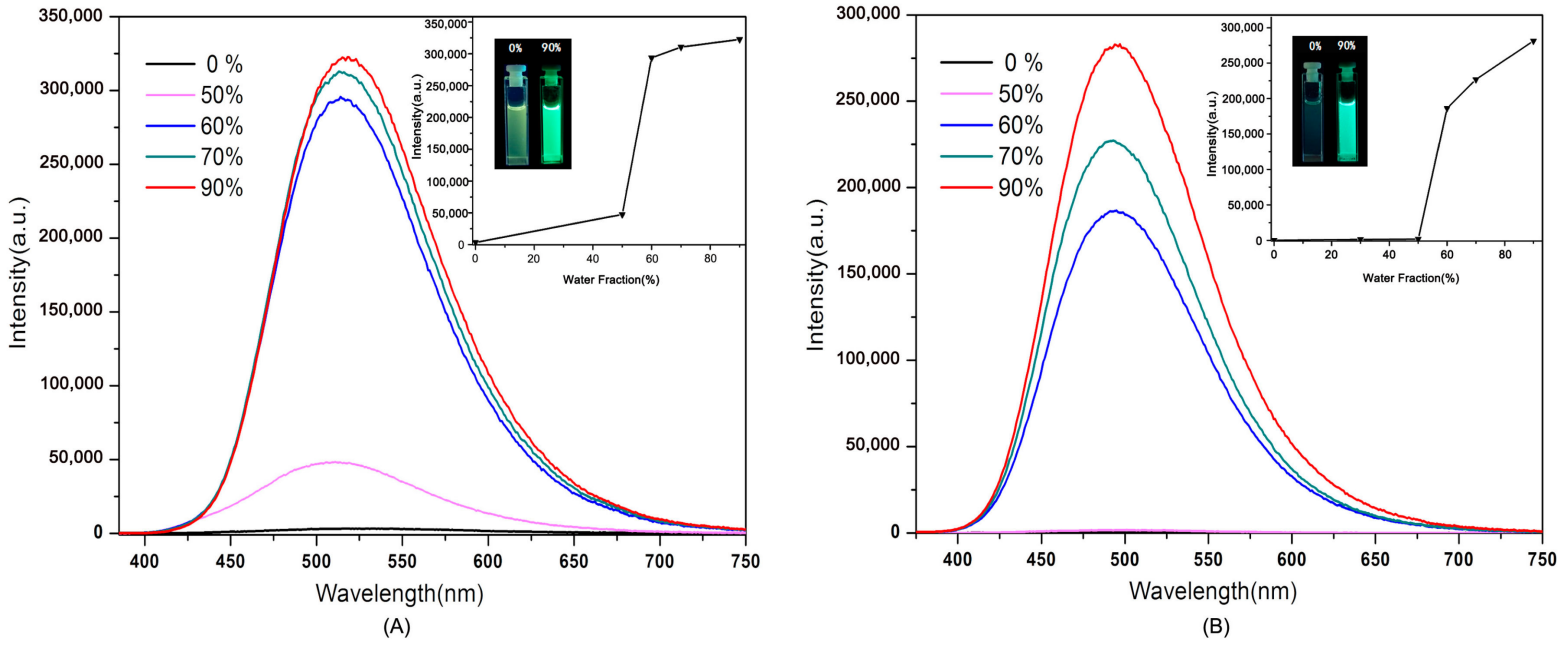
Fluorescence emission spectra of (**A**) **L^a^** (λ_ex_ = 365 nm); (**B**) **L^b^** (λ_ex_ = 343 nm) with inserts showing the plots of maximum emission intensity of ligands versus H_2_O fraction in H_2_O/DMSO mixtures and photographs of assemblies in pure DMSO and 90% H_2_O fractions mixture solution upon excitation at 365 nm using an UV lamp. c_L_ = 50.00 µM.

Photoluminescence study on the ligands (Figure 3) confirmed their AIE nature. In both cases, negligible emissions have been measured in dilute DMSO solution (50.00 µM). However, when water was introduced, the emission intensity for both ligands showed great enhancement. Meanwhile, the quantum yield of **L^a^** exhibited a low value (Φ_F_ = 0.74%) and increased remarkably to 84.2% in the solid state. Similarly, the quantum yield of **L^b^** was too low to be measured under dilute conditions, but increased to 74.1% in the solid state.

**Figure 4:**
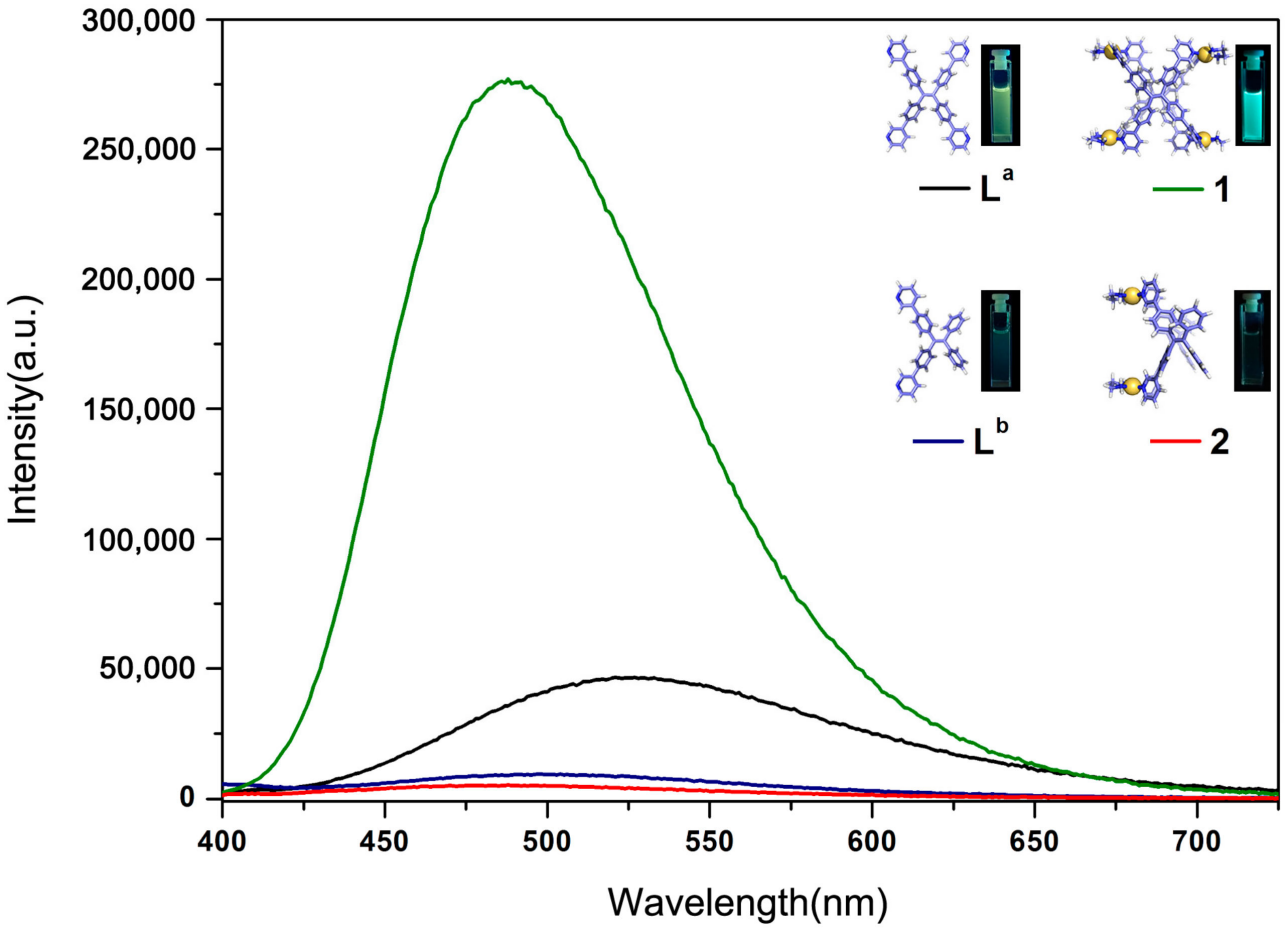
Fluorescence emission spectra of ligand **L^a^** (λ_ex_ = 365 nm), **L^b^** (λ_ex_ = 343 nm) and assemblies **1** (λ_ex_ = 372 nm) and **2** (λ_ex_ = 358 nm) in H_2_O/acetone (*v*:*v* = 1:1) at the same ligand concentration of 50 µM.

Coordination-enhanced emission was confirmed after assembling **L^a^** with the (en)Pd(NO_3_)_2_ clips (Figure 4). Compared to **L^a^**, the four arms on the TPE units of **1** are fixed by the (en)Pd(NO_3_)_2_ units, which forces the two ligands to stack together, thus fully restricting the intramolecular rotations. Though palladium is a well-known fluorescence quencher, such a forced aggregation state induced a remarkable 6-fold fluorescence enhancement over **L^a^** for complex **1** in dilute solution. The ligands on complex **1** are more distorted than its free extended state and the perpendicular conformation decreased the conjugation, resulting that the emission maximum undergoing a blue shift from 528 to 488 nm. These results imply that a coordination-driven self-assembly approach is effective for restricting the intermolecular rotations of the TPE units, thus attenuating the non-radiative decay pathway and turning on the fluorescence. However, ligand **L^b^** has two dangling phenyl rings that remain unrestricted even after assembling with (en)Pd(NO_3_)_2_. The assembled complex **2** shows lower emission intensity than **L^b^** in dilute solution, probably due to the intramolecular motion [30] and the emission quenching effect of the palladium centers.

**Figure 5:**
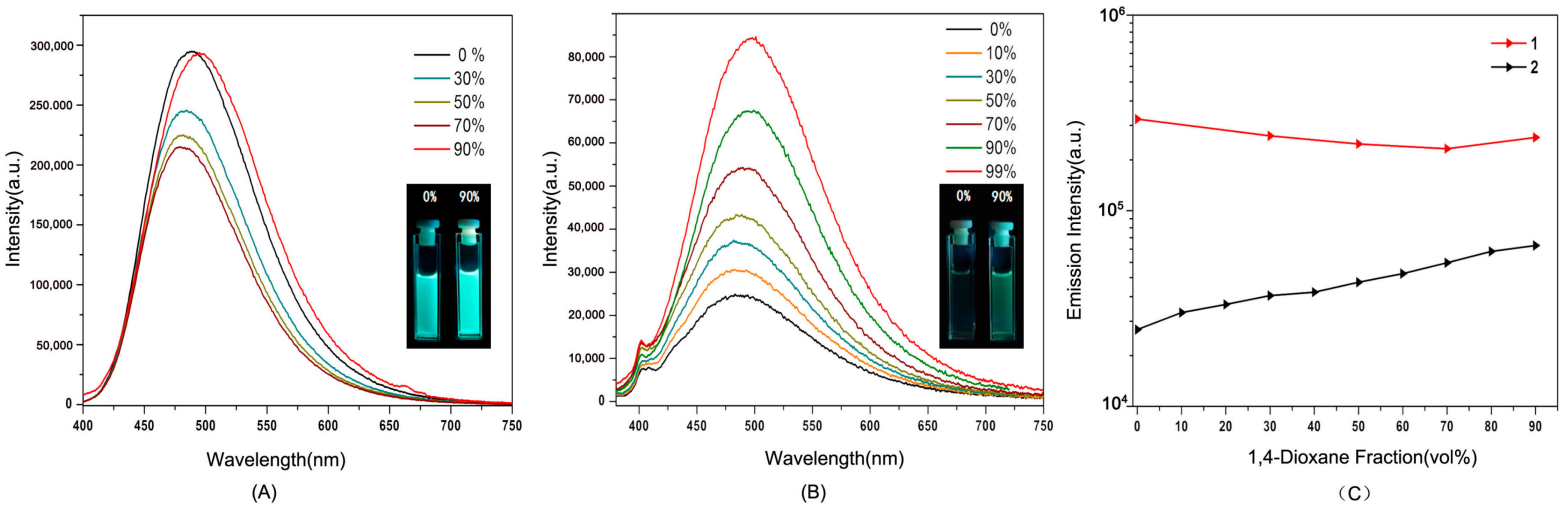
Fluorescence emission spectra of (**A**) **1**, λ_ex_ = 372 nm, (**B**) **2**, λ_ex_ = 358 nm, and (**C**) the plots of maximum emission intensity of assemblies **1**, **2** versus 1,4-dioxane fraction in H_2_O/acetone/1,4-dioxane mixtures. Insert: photographs of assemblies in H_2_O/acetone (*v*:*v* = 1:1) and 90% 1,4-dioxane fractions mixture solution upon excitation at 365 nm using an UV lamp. c_L_ = 50.00 μM.

To examine whether the complexes **1** and **2** are still AIE active, the emission spectra of the assemblies were measured in H_2_O/acetone/1,4-dioxane mixed solvent systems (Figure 5), in which H_2_O/acetone(*v*:*v* = 1:1) is good solvent and 1,4-dioxane is poor solvent. For complex **2**, the emission intensity remains low in H_2_O/acetone solution with no quantum yield detectable, which indicates that free rotation on the TPE units is still possible on the assembly in solution state. With the addition of 1,4-dioxane, the emission intensity gradually increased. Upon increasing the 1,4-dioxane fraction to 99%, remarkably enhanced fluorescence was observed, with a quantum yield determined to be 4.13%. In clear contrast, complex **1** remains strongly emissive in dilute solution, with low responsiveness to the fraction of 1,4-dioxane. Quantum yield (Φ) for **1** was determined to be 4.22% in dilute solution (Figure S25) and increased to 6.57% in solid state. These results clearly confirmed the forced AIE properties by coordination self-assembly.

## 3. Materials and Methods

### 3.1. General

Unless otherwise noted, all chemicals and solvents were purchased from commercial corporations and used without further purification. Deuterated solvents were purchased from Admas (Shanghai, China) and J&K scientific Co. (Beijing, China). 1D- and 2D-NMR spectra were measured on a Bruker Biospin Avance III (400 MHz) spectrometer (Bruker Biospin, Rheinstetten, Germany). ^1^H-NMR chemical shifts were determined with respect to residual solvent signals of the deuterated solvents used. ESI-TOF mass spectra were recorded on an Impact II UHR-TOF mass spectrometry from Bruker (Bruker Biospin, Rheinstetten, Germany). Data analyses and simulation of ESI-TOF mass spectra were processed on a Bruker Data Analysis software (Version 4.3). Molecular modelings were performed on Marerials Studio 6.0 (Accelrys Co., San Diego, CA, USA). UV-Visible absorption spectra were measured on a UV-2700 spectrophotometer (SHIMADZU, Kyoto, Japan). Fluorescent spectra were measured on a FS5 spectrofluorometer (Edinburgh Instruments Ltd., Livingston, UK). The emission quantum yields in solution and solid state were measured by FS5 Spectra Fluorometer (Edinburgh Instruments Ltd., Livingston, UK) with integrating sphere.

### 3.2. Synthesis Procedure

#### 3.2.1. Synthesis of Ligand **L^a^**

**Scheme 2:**
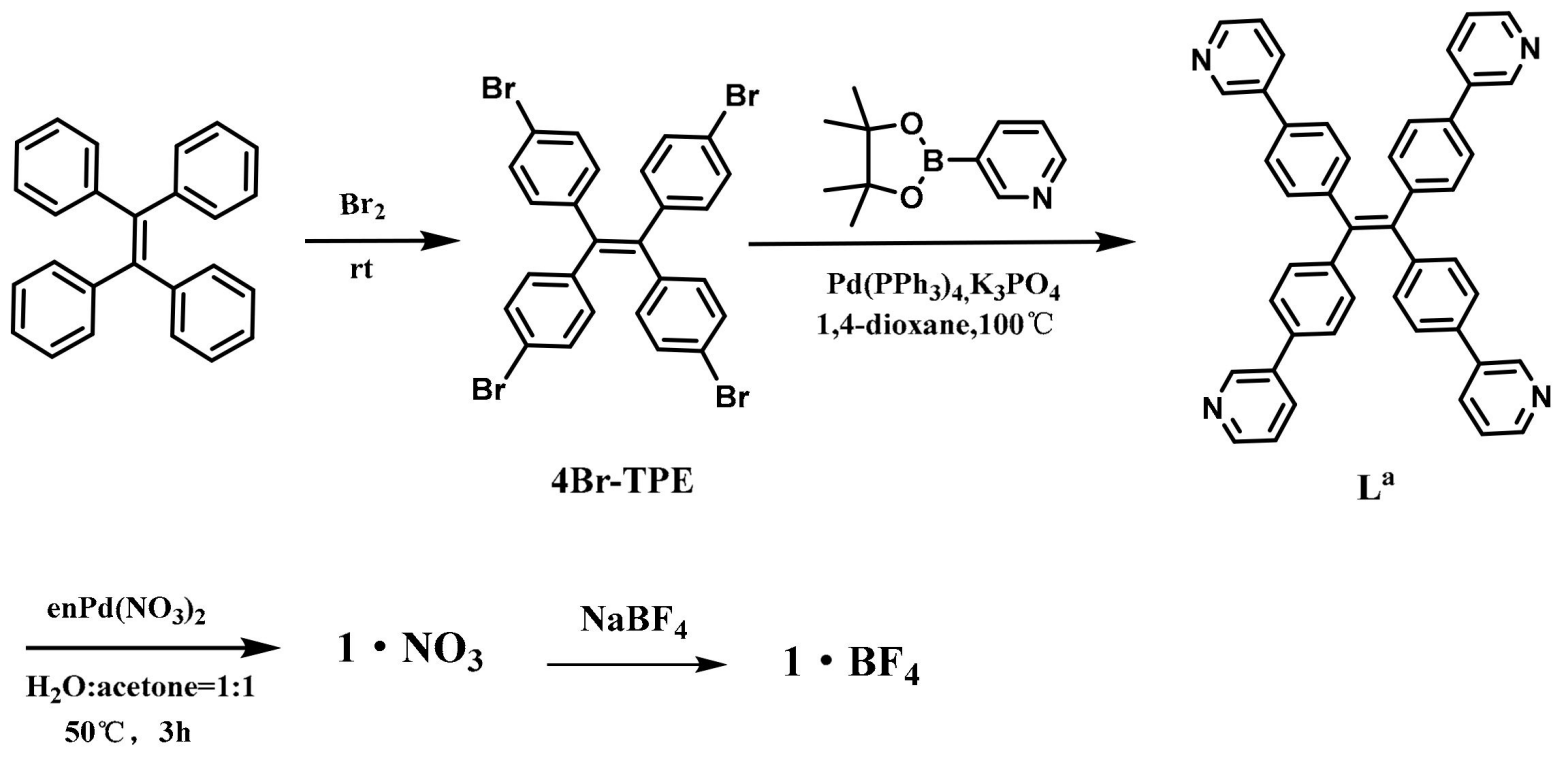
Synthetic procedures of ligand **L^a^** and assembly **1**.

**4Br-TPE** was prepared using reported literature procedures (Scheme 2) [26]. Tetraphenylethene (5 g, 10 mmol) was treated with bromine (7.5 mL, 0.15 mol) and the mixture was kept for 16 h at room temperature. The resulting solid was dissolved in hot toluene (120 mL), then concentrated to 20 mL. The precipitate was filtered and washed with hexane. The product was further purified by chromatography on SiO_2_ (Hexanes/CH_2_Cl_2_, *v*/*v* = 20/1). 5.94 g (9.17 mmol, Yield: 61%) of a colorless solid was obtained. ^1^H-NMR (400 MHz, DMSO-*d*_6_): δ 7.39 (d, *J* = 8.3 Hz, 8H), 6.92 (d, *J* = 8.3 Hz, 8H). ^13^C-NMR (100 MHz, DMSO-*d*_6_) δ 141.50 (s), 139.38 (s), 132.79 (s), 131.19 (s), 120.48 (s).

**L^a^** was prepared using reported literature procedures [27,28]. **4Br-TPE** (161 mg, 0.25 mmol), 3-(4,4,5,5-tetramethyl-1,3,2-dioxaborolan-2-yl)pyridine (246 mg, 1.2 mmol), Pd(PPh_3_)_4_ (40 mg, 0.035 mmol) and K_3_PO_4_ (1.1 g, 5.18 mmol) were added into a 100 mL three-necked flask. Under N_2_ atmosphere, 1,4-dioxane (50 mL) was added. The suspension was stirred at 100 °C for 3 d. The mixture was evaporated and extracted with chloroform and water. The organic phase was collected and dried over MgSO_4_ and evaporated. The crude product was purified by silica gel column chromatography (CH_2_Cl_2_:CH_3_OH = 100:1). 64 mg (0.1 mmol, Yield: 40%) of a yellow green powder was obtained. ^1^H-NMR (400 MHz, CDCl_3_): δ 8.83 (d, *J* = 1.5 Hz, 4H), 8.56 (d, *J* = 3.4 Hz, 4H), 7.85 (d, *J* = 7.9 Hz, 4H), 7.42 (d, *J* = 8.2 Hz, 8H), 7.33 (dd, *J* = 7.8, 4.8 Hz, 4H), 7.23 (d, *J* = 8.2 Hz, 8H). ^13^C-NMR (100 MHz, CDCl_3_) δ 148.52 (s), 148.18 (s), 143.26 (s), 140.60 (s), 136.11 (s), 135.95 (s), 134.10 (s), 132.16 (s), 126.57 (s), 123.54 (s).

#### 3.2.2. Synthesis of Assembly **1**

Ligand **L^a^** (4.72 mg, 7.36 μmol) was treated with (en)Pd(NO_3_)_2_ (4.7 mg,16.2 μmol) in a mixture solution of H_2_O and acetone (*v*:*v* = 1:1) and stirred at 50 °C for 3 h. **1·NO_3_** was obtained. ^1^H-NMR (400 MHz, D_2_O:acetone-*d*_6_ = 1:1): δ 9.65 (s, 4H), 9.03 (s, 4H), 8.41 (s, 4H), 7.84 (s, 4H), 7.72 (s, 8H), 7.42 (s, 8H), 3.12 (s, 8H). ^13^C-NMR (100 MHz, D_2_O:acetone-*d*_6_ = 1:1): δ 213.88 (s), 151.20 (s), 149.24 (s), 144.23 (s), 141.47 (s), 139.20 (s), 138.65 (s), 134.48 (s), 132.34 (s), 127.80 (s), 127.14 (s), 47.30 (s). 

To the solution of **1·NO_3_** was added excess NaBF_4_ aqueous solution and stirred for ten minutes. The precipitate was filtered and washed with water. **1·BF_4_** was obtained and characterized by ESI-TOF-MS: *m*/*z* 573.6163 {[(en)_4_Pd_4_**L^a^**_2_(BF_4_)_4_]^4+^}, 793.8219 {[(en)_4_Pd_4_**L^a^**_2_(BF_4_)_5_]^3+^}, 1234.2355 {[(en)_4_Pd_4_**L^a^**_2_(BF_4_)_6_]^2+^}.

#### 3.2.3. Synthesis of Ligand **L^b^** and Assembly **2**

**Scheme 3:**
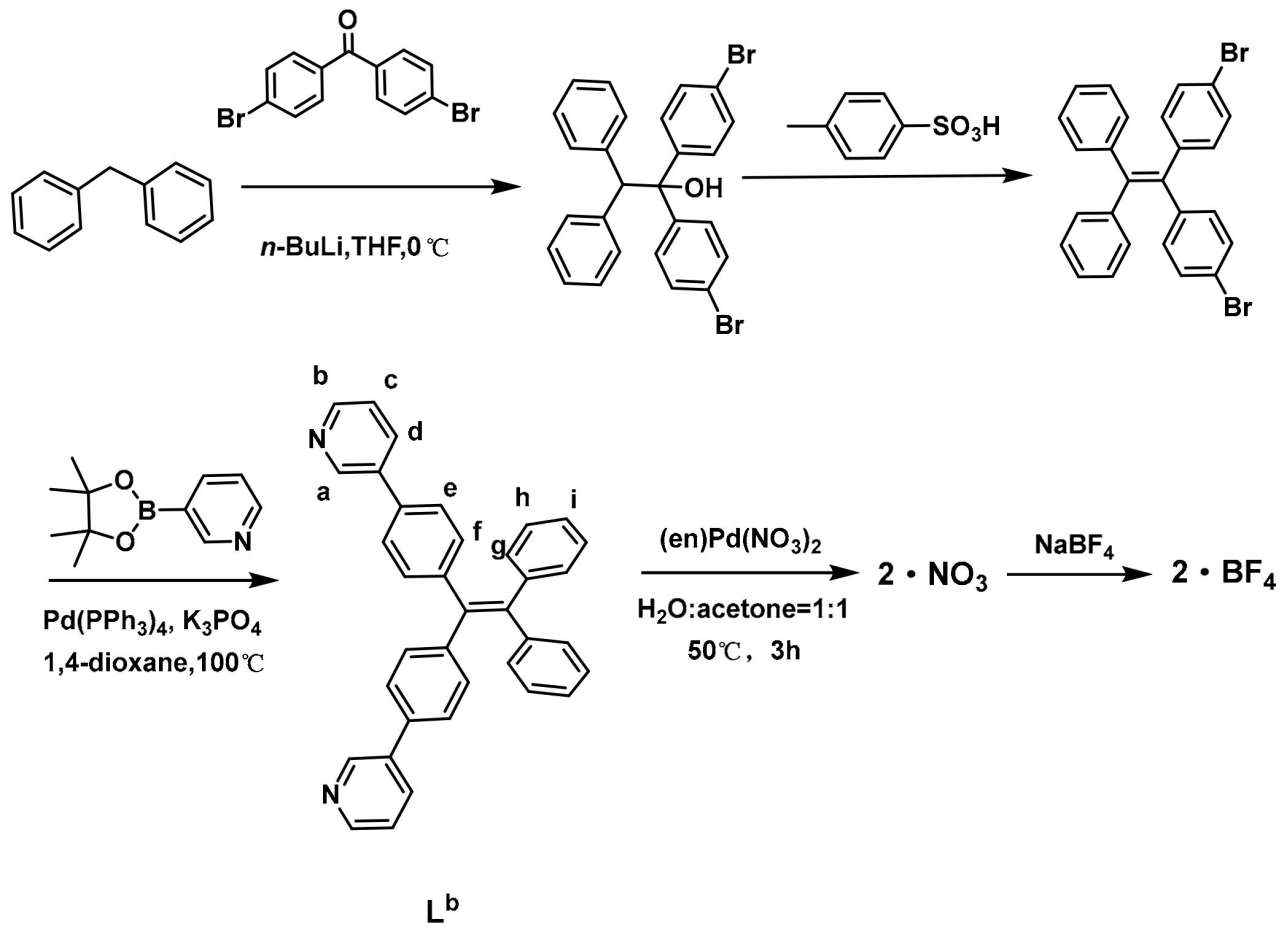
Synthetic procedures of ligand **L^b^** and assembly **2**.

The preparation of **L^b^** followed the literature procedure (Scheme 3) [21] and the assembly of **L^b^** with (en)Pd(NO_3_)_2_ under the similar procedure above gave complex **2·BF_4_**. ^1^H-NMR (400 MHz, CD_3_CN): δ 9.12 (s, 2H), 8.75 (d, *J* = 5.0 Hz, 2H), 8.12 (d, *J* = 8.0 Hz, 2H), 7.77–7.55 (m, 2H), 7.48 (d, *J* = 8.0 Hz, 4H), 7.22 (d, *J* = 8.0 Hz, 4H), 7.15 (s, 6H), 7.09 (d, *J* = 6.8 Hz, 4H), 4.14 (s, 4H), 2.81 (s, 4H). ^13^C-NMR (100 MHz, CD_3_CN) δ 150.785 (s), 150.741(s), 145.69 (s), 144.27 (s), 144.13 (s), 140.08 (s), 139.61 (s), 138.98 (s), 133.99 (s), 133.09 (s), 131.81 (s), 128.95 (s), 127.98 (s), 127.71 (s), 127.46 (s), 47.62 (s). ESI-TOF-MS: *m*/*z* 326.5877 {[(en)_2_Pd_2_**L^b^**_2_]^4+^}, 464.4514 {[(en)_2_Pd_2_**L^b^**_2_(BF_4_)_1_]^3+^}, 740.1784 {[(en)_2_Pd_2_**L^b^**_2_(BF_4_)_2_]^2+^}.

## 4. Conclusions

In summary, fluorescent supramolecular coordination complexes have been self-assembled from TPE-based polypyridine ligands and *cis*-blocked Pd^2+^ corners. Distinctively, when the four arms of the TPE units are fixed, the assembly remains strongly emissive not only in dilute solution but also in the aggregated state. We envisage that this coordination-enhanced emissive supramolecule may find applications in palladium sensors, biomedical sensing, or labeling.

## Supplementary Materials

The following supplementary materials are available online: synthesis, NMR spectra, ESI-TOF-MS, UV-Vis spectra and fluorescence spectra of ligands and assemblies.
