# Supplementary material for: Coordination-Enhanced Luminescence on Tetra-Phenylethylene-Based Supramolecular Assemblies
Source: Molecules. 2018 Feb 9;23(2):363. doi: 10.3390/molecules23020363 (PMC6017277; doi:10.3390/molecules23020363)
Supplement: Supplementary file 1 [file molecules-23-00363-s001.pdf]

Supporting Materials

Coordination-Enhanced Luminescence on Tetraphenylethylene-Based Supramolecular Assemblies

Qian-Qian Yan, Shao-Jun Hu, Guang-Lu Zhang, Ting Zhang, Li-Peng Zhou, and Qing-Fu Sun*

Corresponding Email: qfsun@fjirsm.ac.cn

Contents

1. Supporting Figures and Tables

1.1 NMR spectra

1.2 DOSY spectra

1.3 ESI-TOF-MS

1.4 UV-vis absorption spectra

1.5 Fluorescence spectra

1. Supporting figures and tables

1.1 NMR spectra and ESI-TOF mass spectra

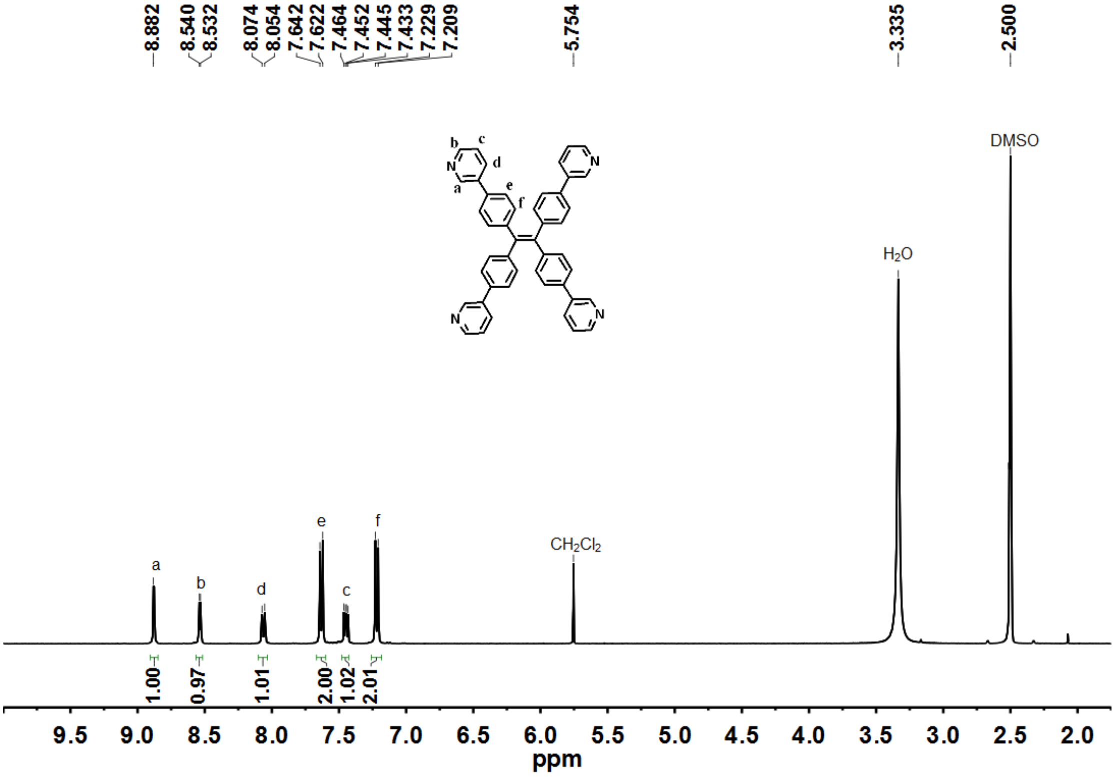

Fig S1. ^{1}H NMR spectrum of the ligand **L^a^** (400 MHz, DMSO- *d*$_6$, 298K)

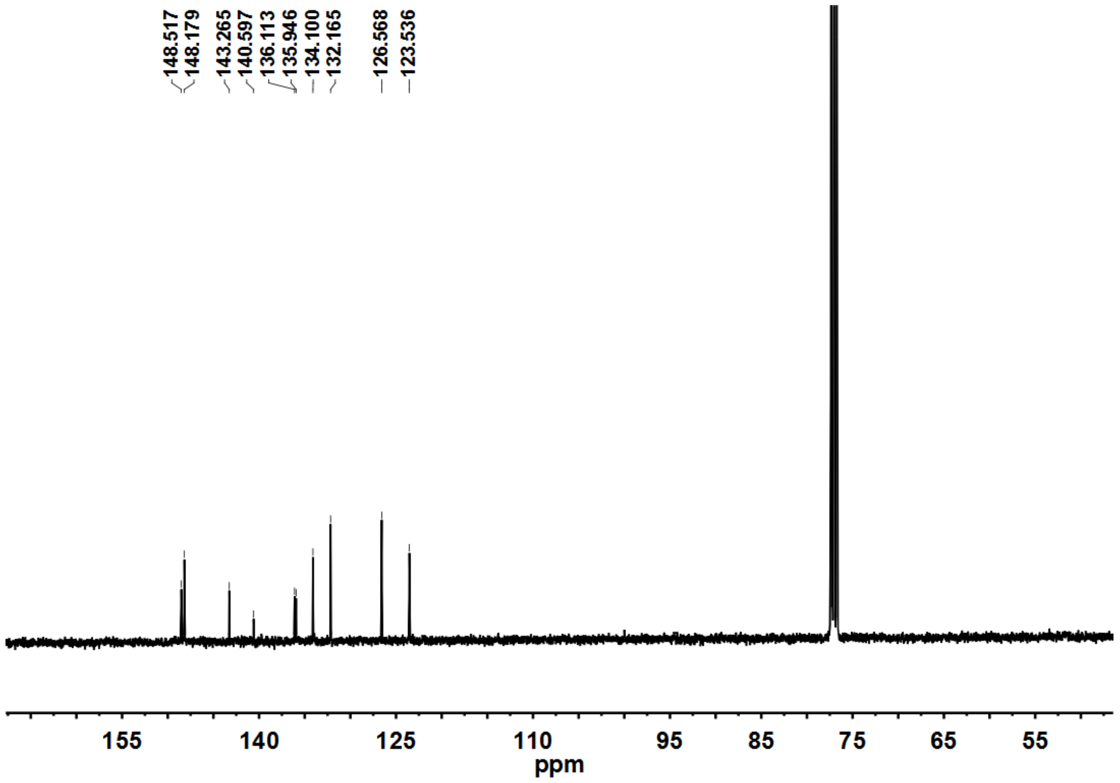

Fig S2. ^{13}C NMR spectrum of the ligand **L^{a}** (100 MHz, $CDCl_3$, 298K)

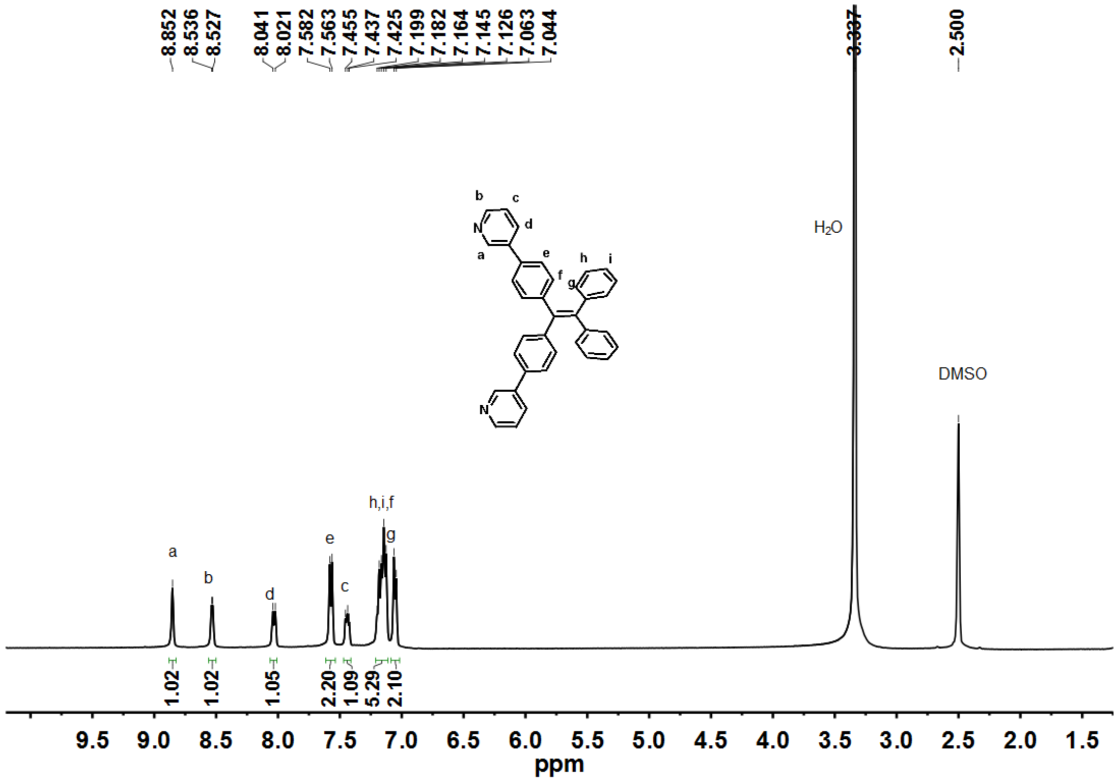

Fig S3. ^{1}H NMR spectrum of the ligand **L^{b}** (400 MHz, DMSO-d_6, 298K)

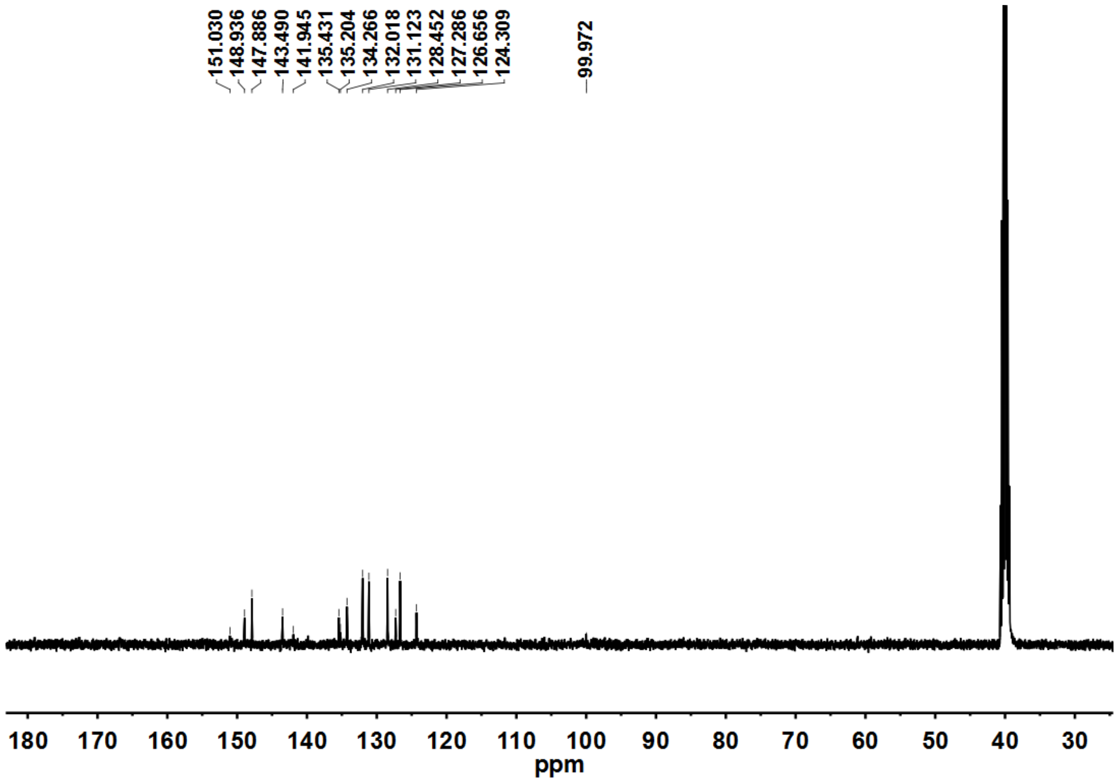

Fig S4. ^{13}C NMR spectrum of the ligand $\mathbf{L^b}$ (100 MHz, DMSO-d_6, 298K)

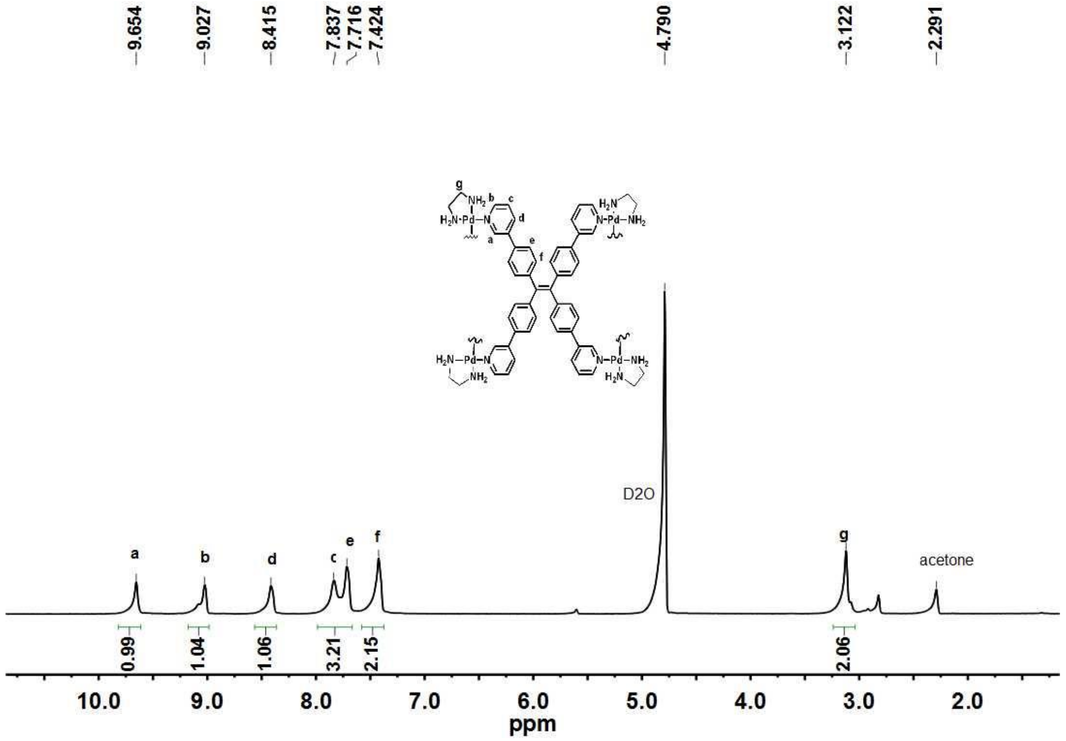

Fig S5. ^{1}H NMR spectrum of the assembly **1** (400 MHz, D_2O: acetone- d_6 =1:1, 298K)

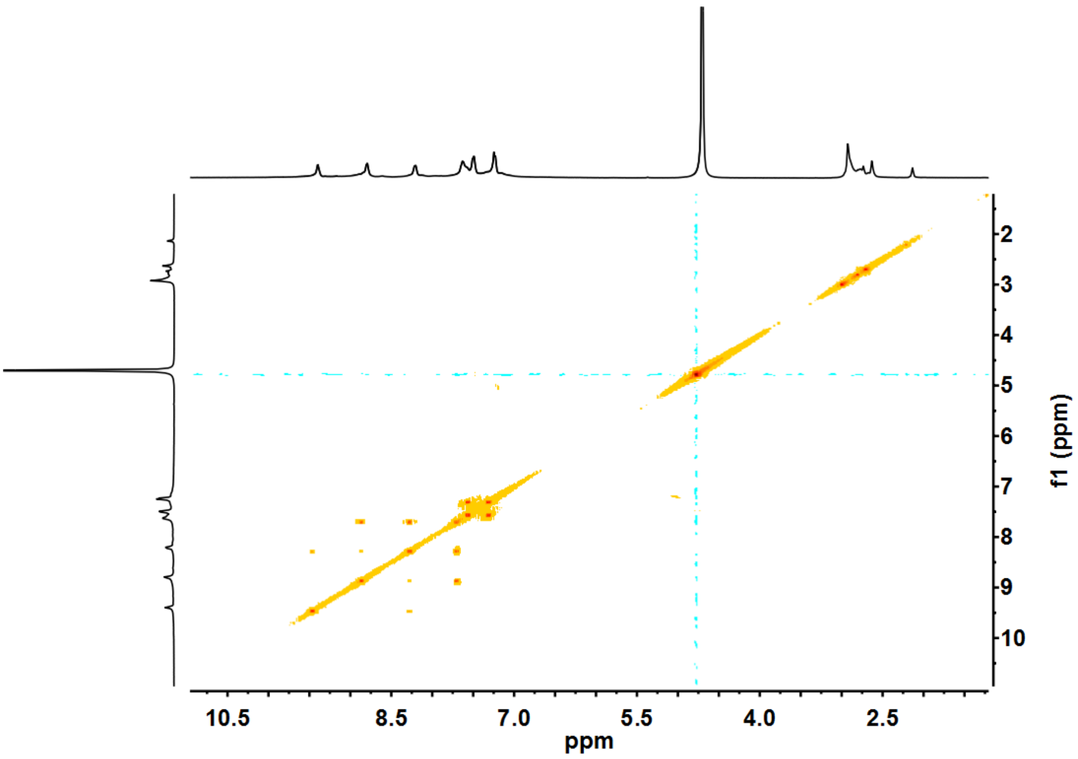

Fig S6. ^{1}H-^{1}H COSY spectrum of the **1·NO$_3$** (400 MHz, D_2O:acetone- d_6 =1:1,298K)

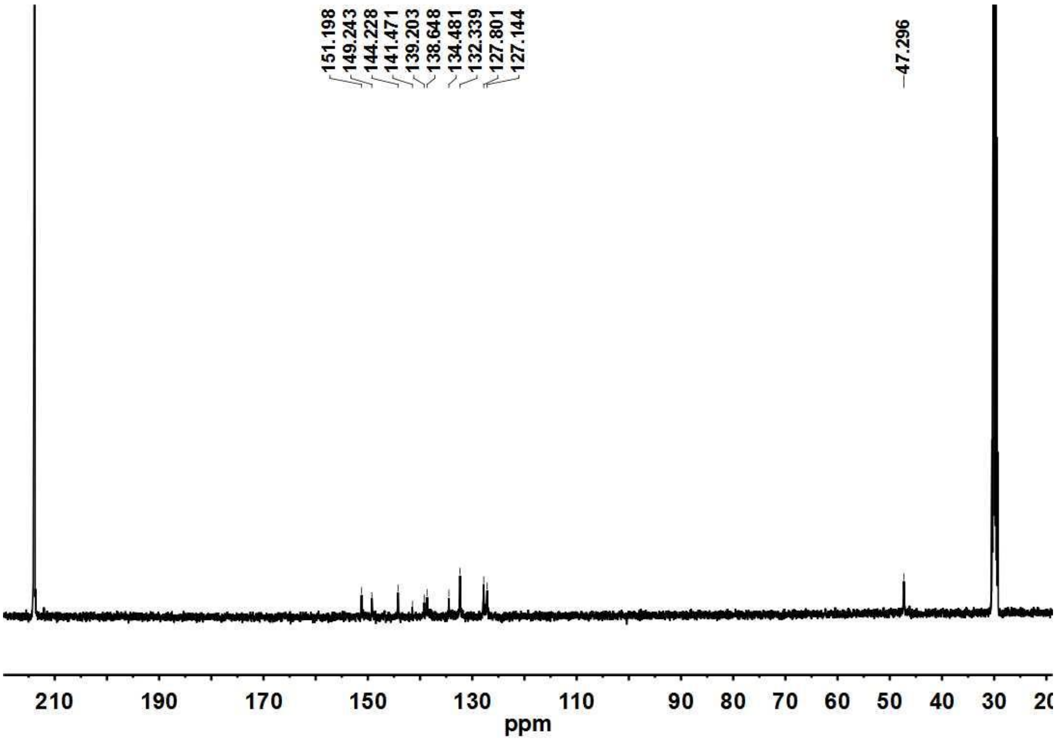

Fig S7. ^{13}C NMR spectrum of the assembly **1** (100MHz, D_2O: acetone-d_6 =1:1, 298K).

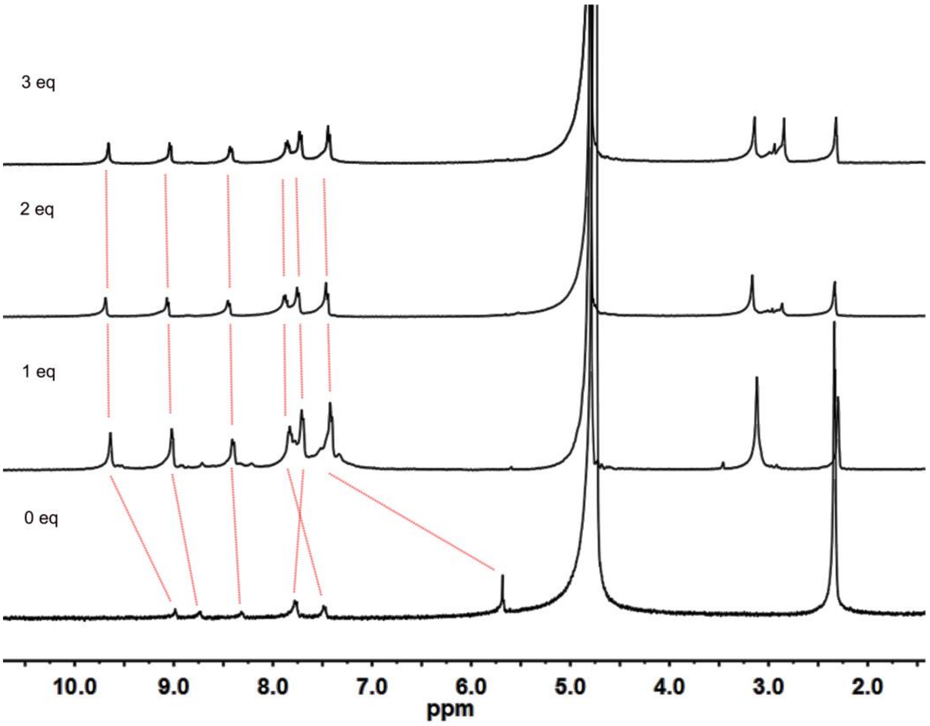

Fig S8. 1H NMR spectra of L[a] with the titration of (en)Pd$(NO_3)_2$ (400MHz, D_2O: acetone-d_6=1:1, 298K).

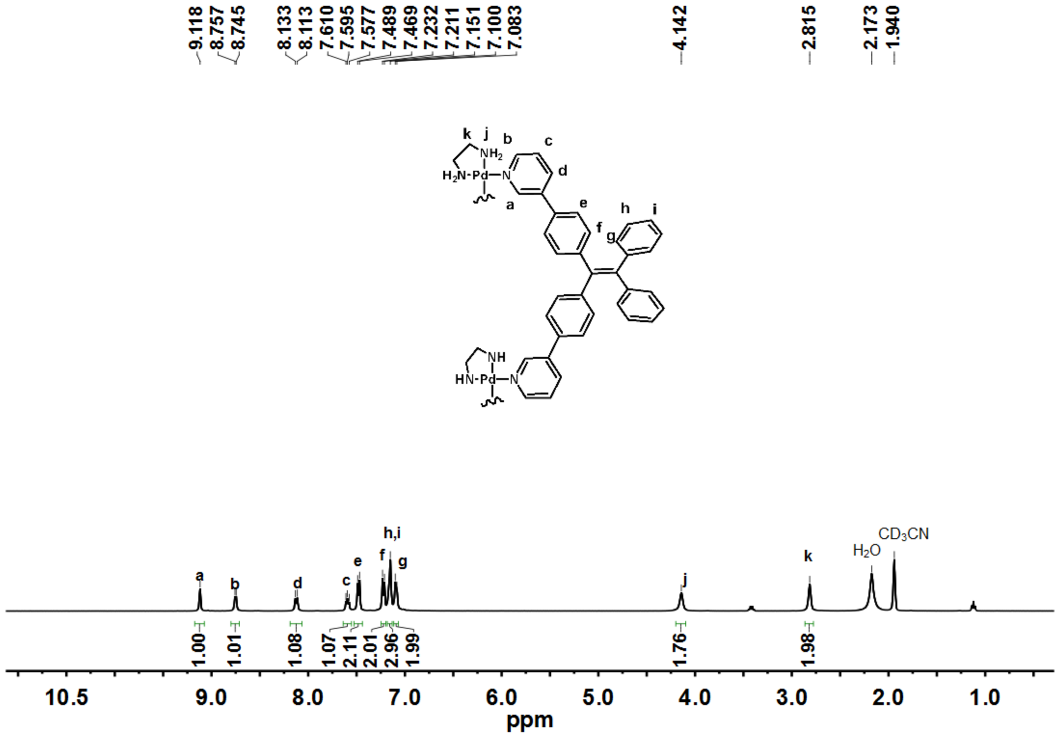

FigS9. ^{1}H NMR spectrum of the assembly **2·BF**$_4$ (400 MHz,CD_3CN, 298K)

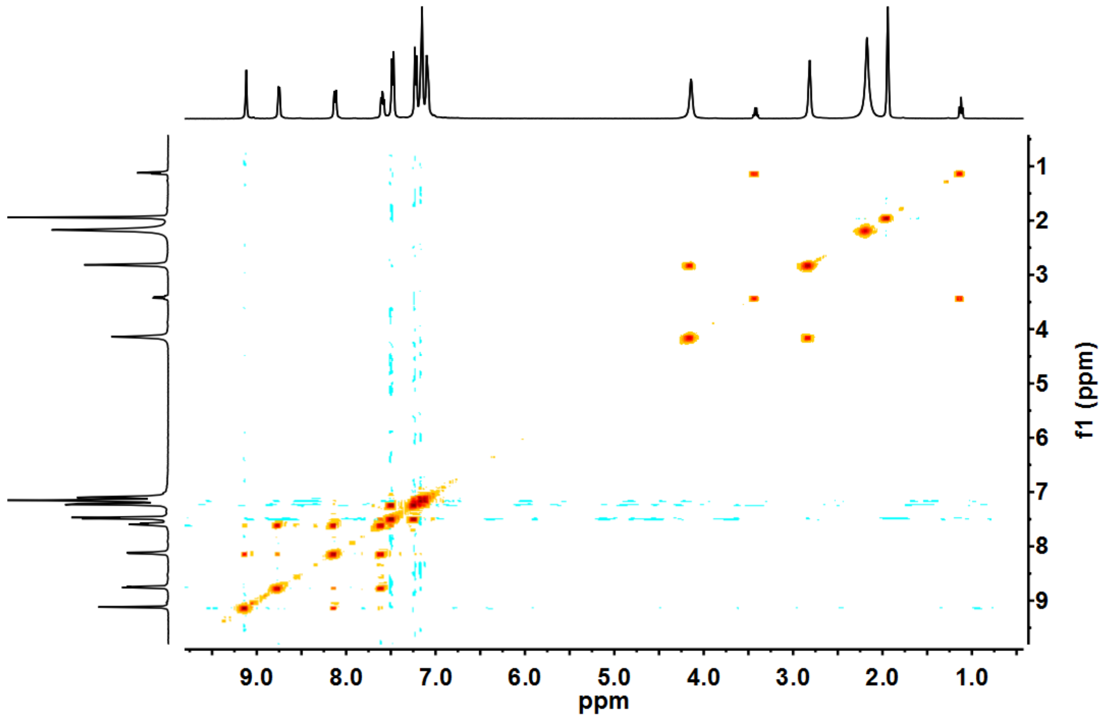

Fig S10. ^{1}H-^{1}H COSY spectrum of the **2·BF$_4$** (400 MHz,CD_3CN, 298K)

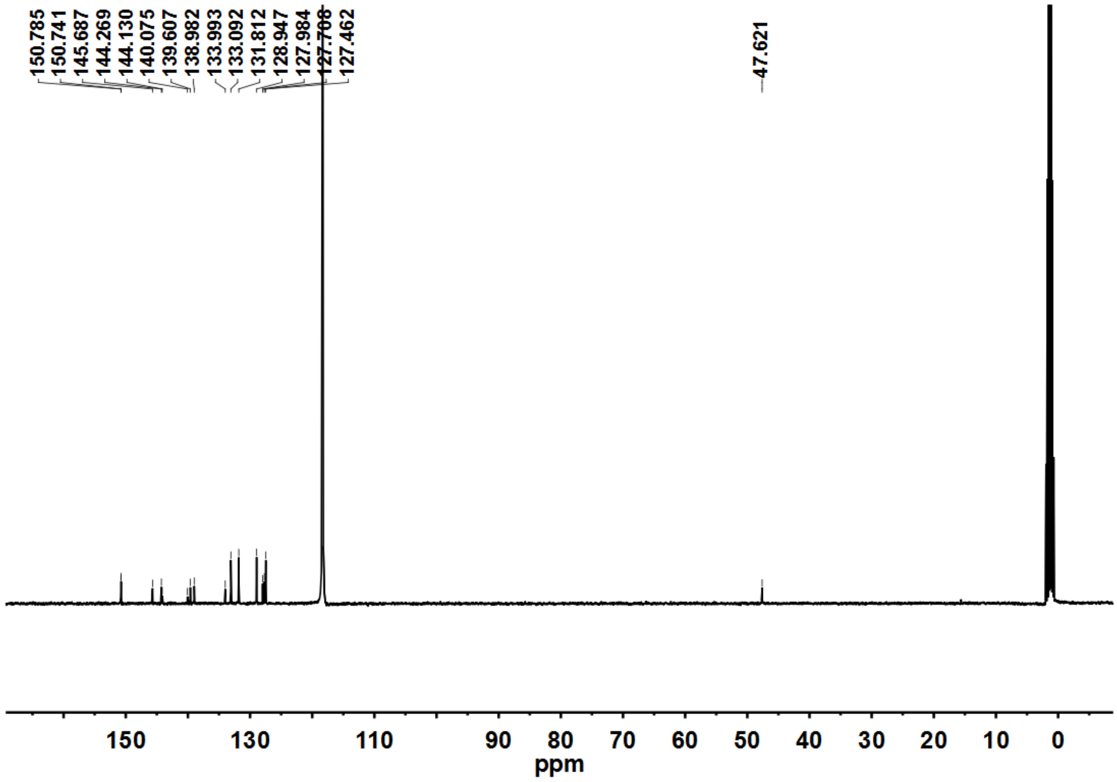

Fig S11. ^{13}C NMR spectrum of the **2·BF$_4$**(100MHz,CD_3CN, 298K)

1.2 DOSY spectra

Stokes-Einstein equation,

$$D = k_B T/6\,\pi\eta r$$

was applied to estimate the dynamic radius for complexes 1 and 2 and of the species resulting from reacting an equivalent mixture of ligand with (en)Pd$(NO_3)_2$.Where D = diffusion coefficient; k_B= Boltzmann'sconstant; T = absolute temperature; η=dynamic viscosity of the solvent (1.2 mPa s, 298K， calculated by Ubbelohde viscometer); r= hydrodynamic radius of aspherical particle.

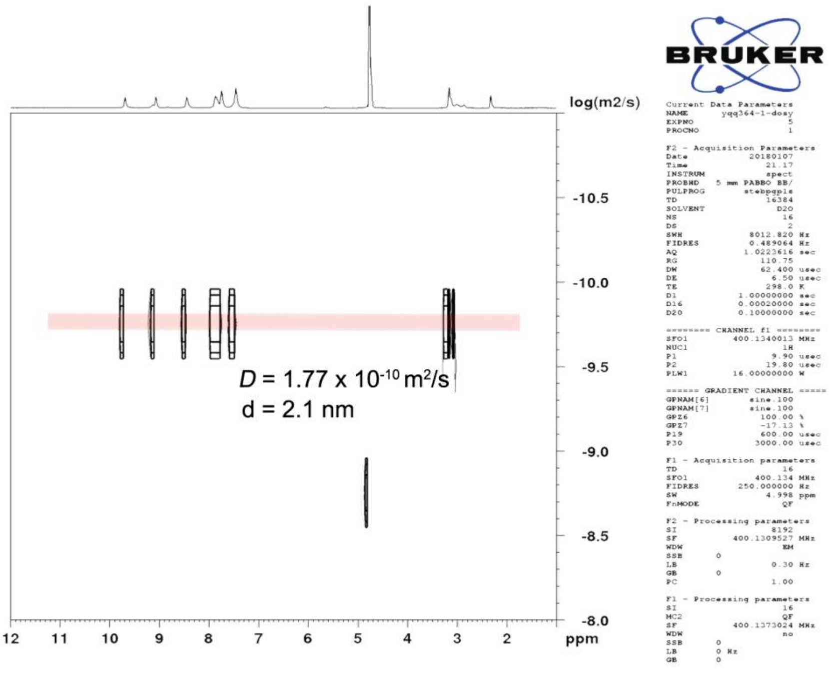

Fig S12. ^{1}H DOSY spectrum of compound **1** (400 MHz, D_2O: acetone-d_6 = 1:1, 298K)

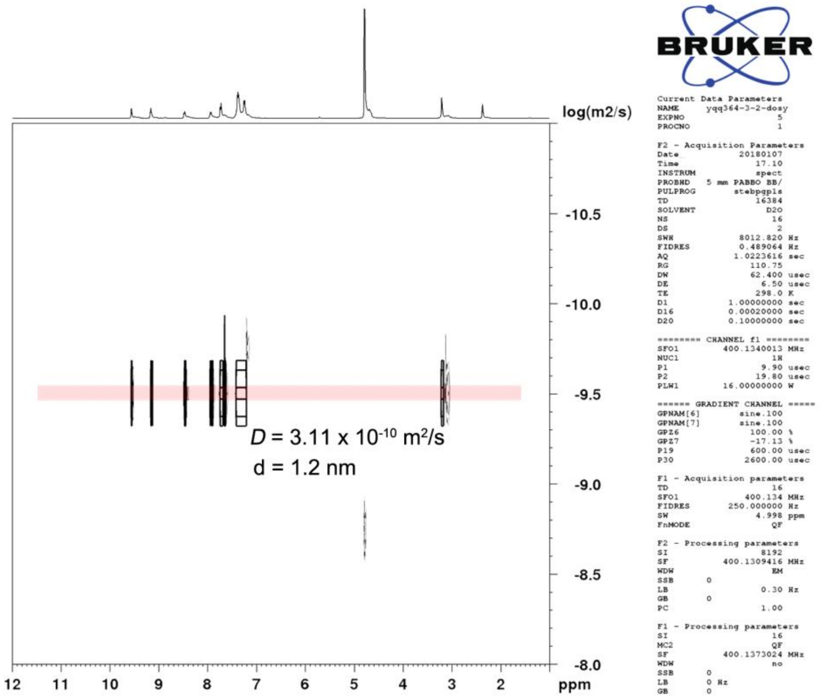

Fig S13. ^{1}H DOSY spectrum of compound **2** (400 MHz, D_2O:acetone-d_6 =1:1, 298K)

1.3 ESI-TOF-MS

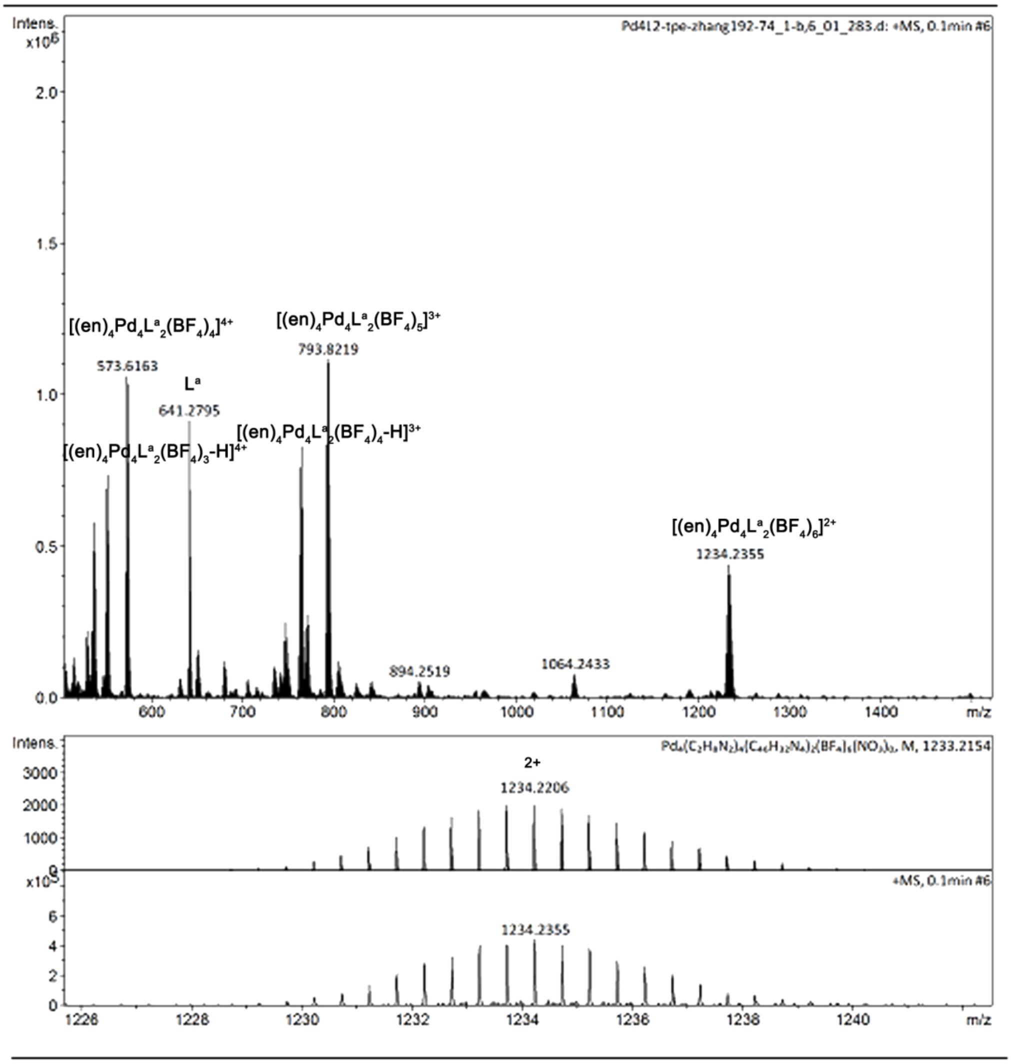

Fig S14. ESI-TOF mass spectrum of **1·BF$_4$** and the observed and simulated isotopic patterns of the 2+ peaks

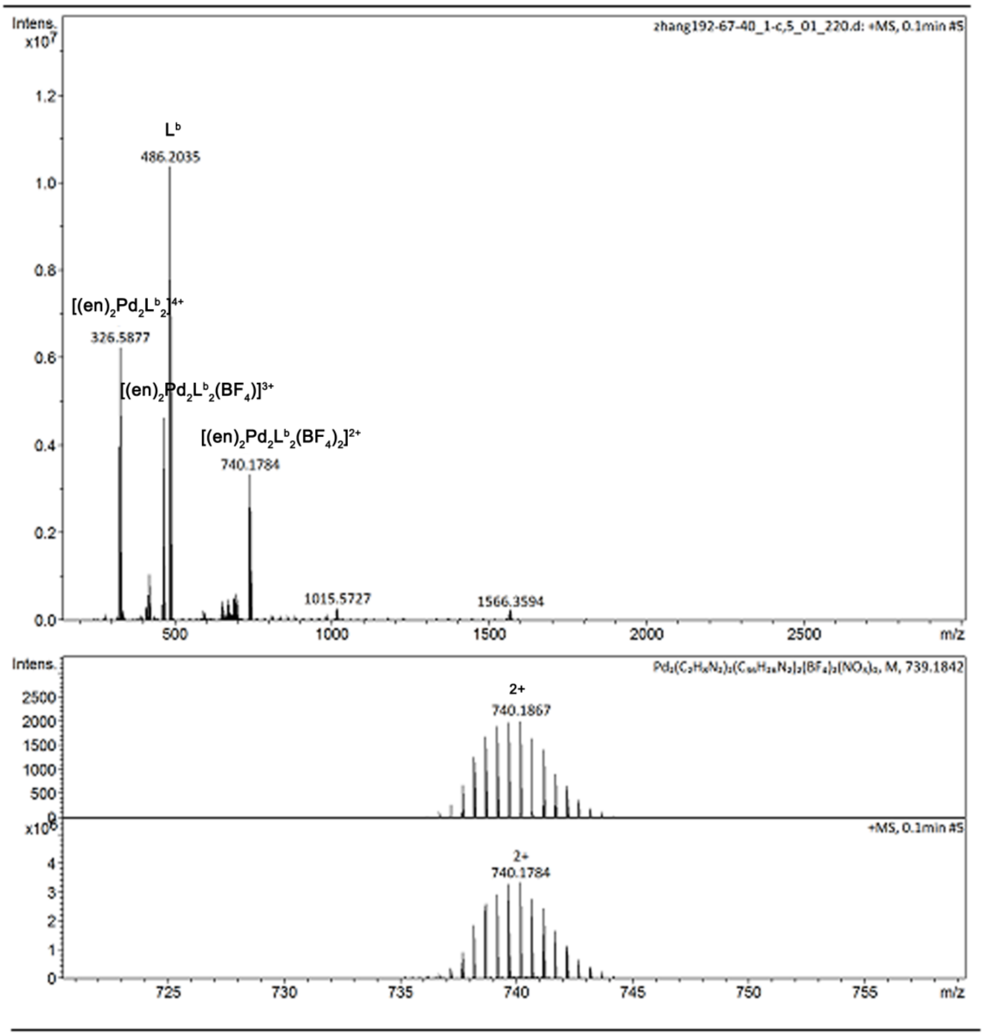

Fig S15. ESI-TOF mass spectrum of **2 ·BF**$_4$ and the observed and simulated isotopic patterns of the 2+ peaks.

1.4 UV-vis absorption spectra

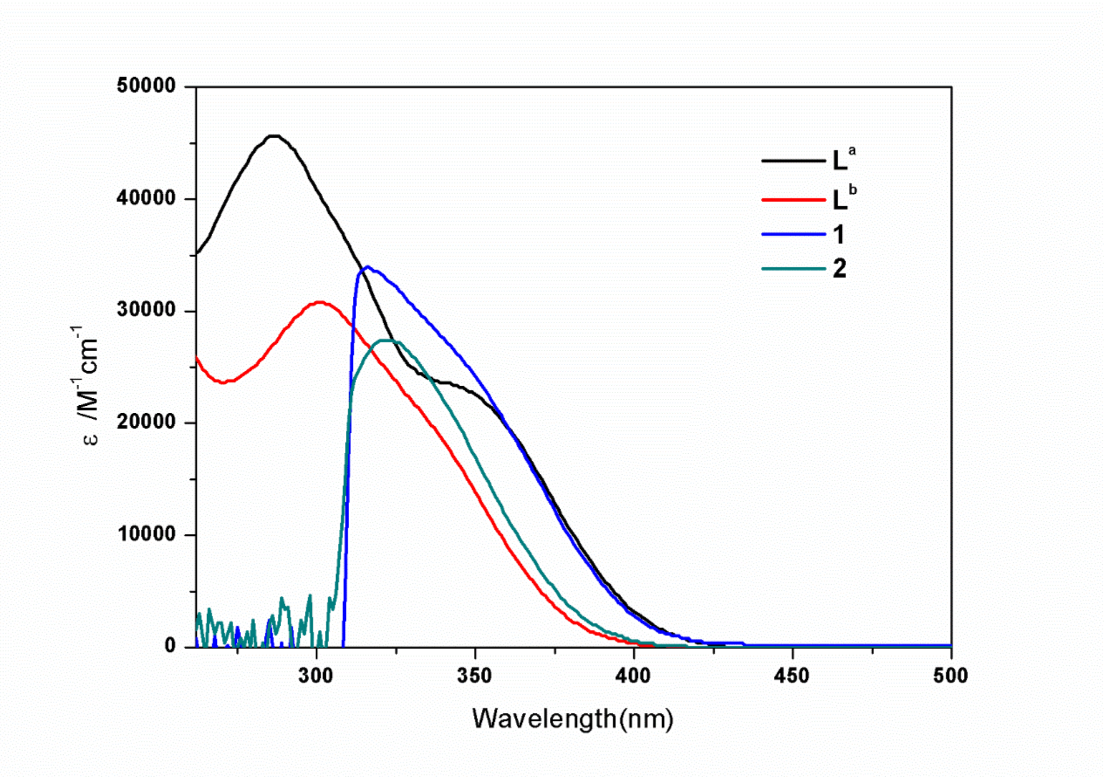

Fig S16. UV-vis absorption spectra of ligand **L^a^**, **L^b^** in DMSO and complexes **1** and **2** in H_2O/ acetone (v:v=1:1). (c_L =50 μM).

1.5 Fluorescence spectra

Table S1. Fluorescence and quantum yield of ligands and assemblies in dilute solution (c_L =50 μM) and in solid state.

	Dilute solution			Solid state		
	λex(nm)	λem(nm)	Φ_F(%)	λex(nm)	λem(nm)	Φ_F(%)
**L^a**	365	528	0.739	408	488	84.2
**L^b**	343	490	-	386	440	74.1
**1**	372	492	4.22	458	543	6.57
**2**	358	488	-	430	528	4.13

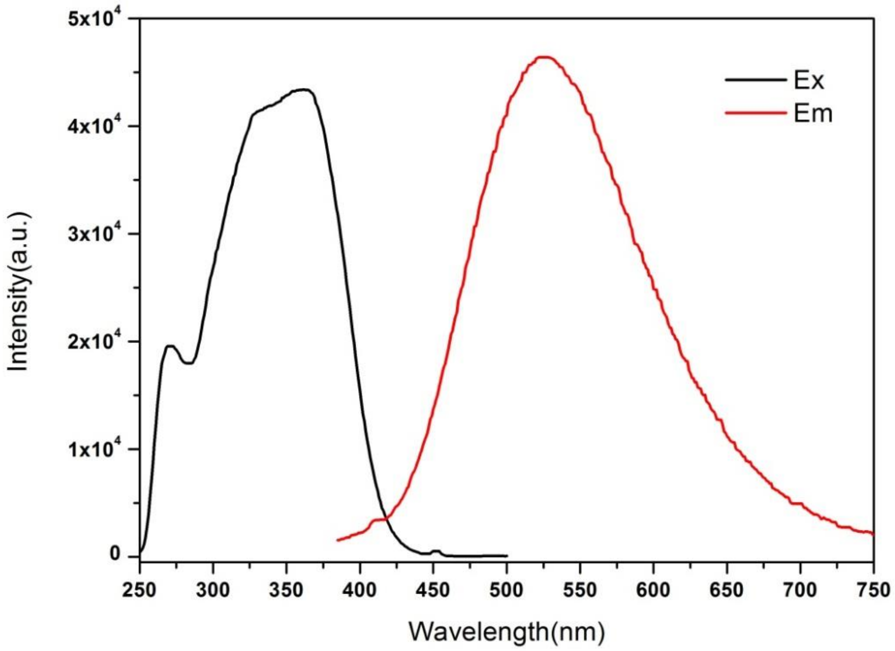

Fig S17. Fluorescence excitation (black, λ_{em} = 528 nm) and emission (red, λ_{ex} = 365nm) spectra of $\mathbf{L^a}$ in DMSO (c_L =50 μM).

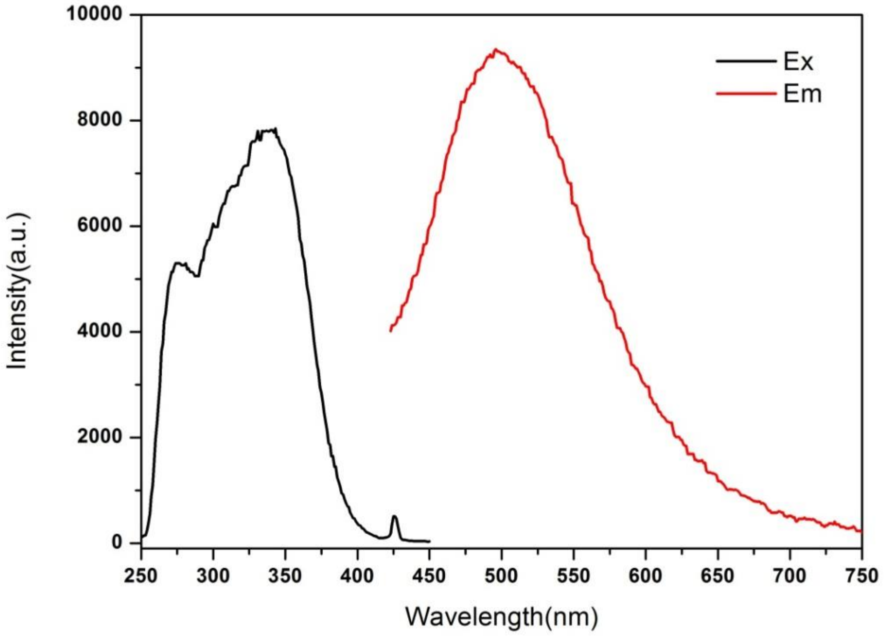

Fig S18. Fluorescence excitation (black, λ_{em} = 490 nm) and emission (red, λ_{ex} = 343nm) spectra of $\mathbf{L^b}$ in DMSO (c_L =50 μM).

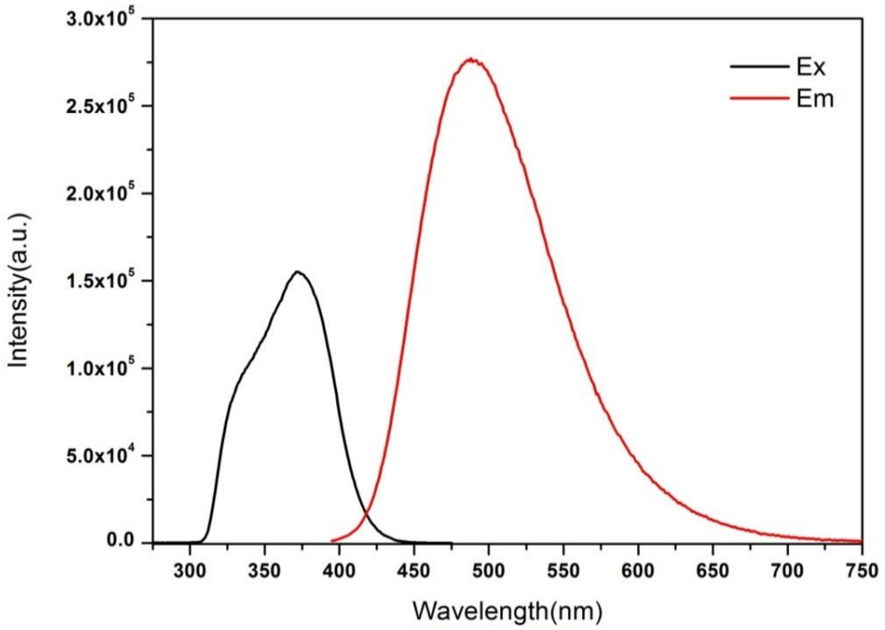

Fig S19. Fluorescence excitation (black, λ_{em} = 492 nm) and emission (red, λ_{ex} = 372nm) spectra of **1** in H_2O/acetone(v:v=1:1) (c_L =50 μM).

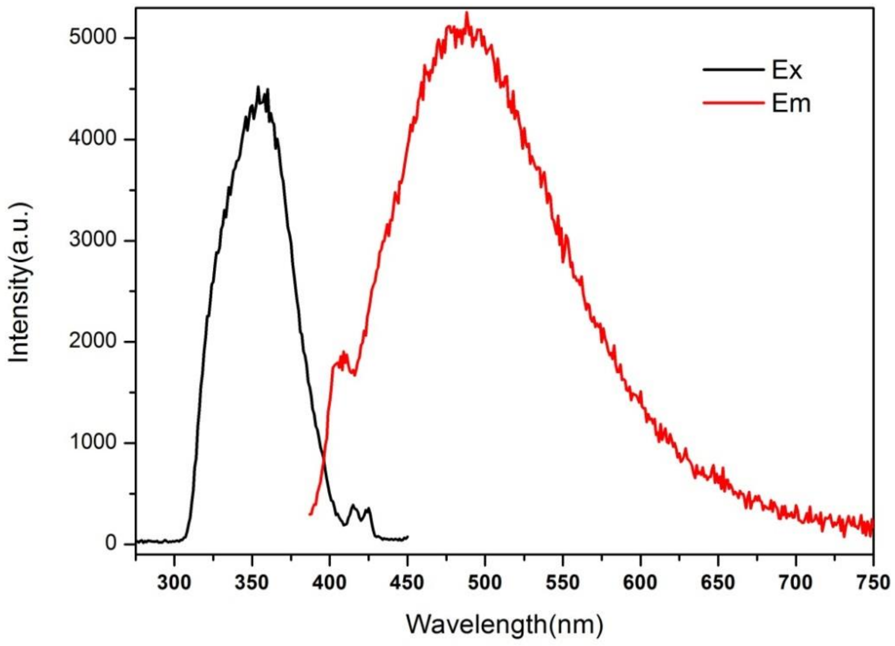

Fig S20. Fluorescence excitation (black, λ_{em} = 488 nm) and emission (red, λ_{ex} = 358 nm) spectra of **2** in H_2O/acetone (v:v=1:1) (c_L =50 μM).

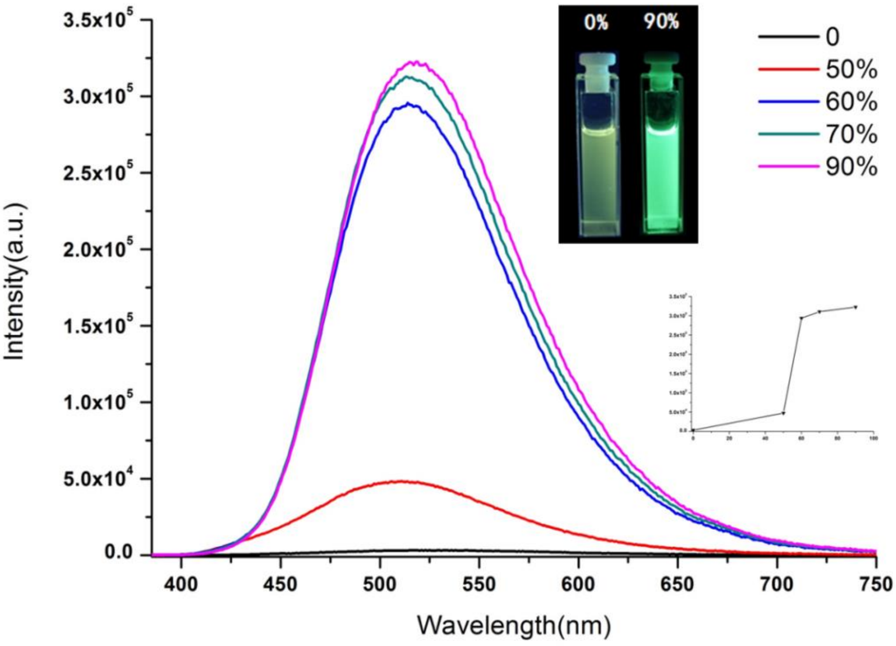

Fig S21. The photograph and emission spectrum of $\mathbf{L^a}$ with increasing H_2O fractions in H_2O/DMSO mixtures (λ_{ex} = 343 nm, c_L = 50 μM).

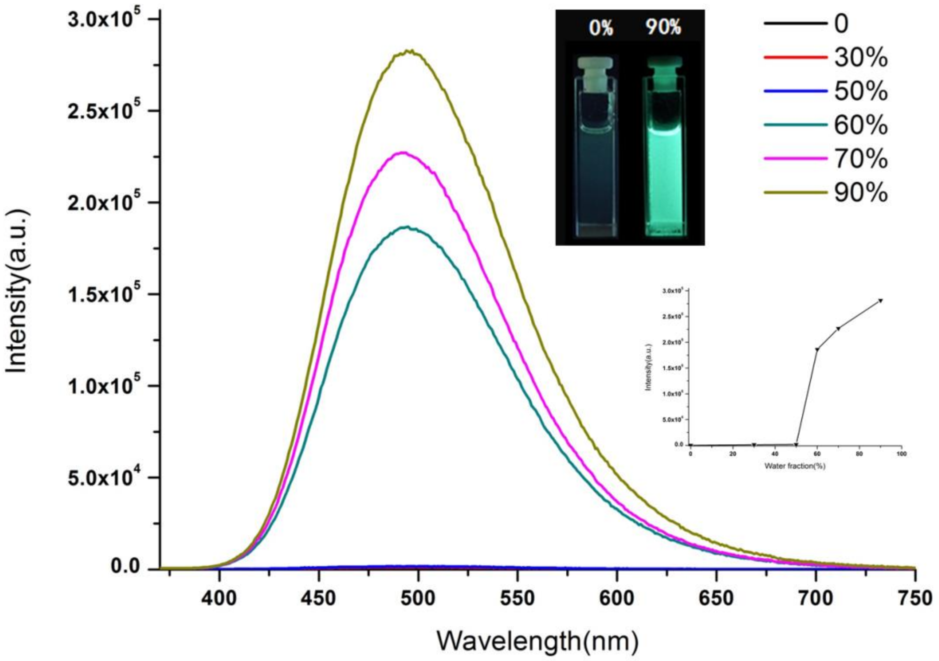

Fig S22. The photograph and emission spectrum of $\mathbf{L^b}$ with increasing H_2O fractions in H_2O/DMSO mixtures. (λ_{ex} = 365 nm, c_L = 50 μM).

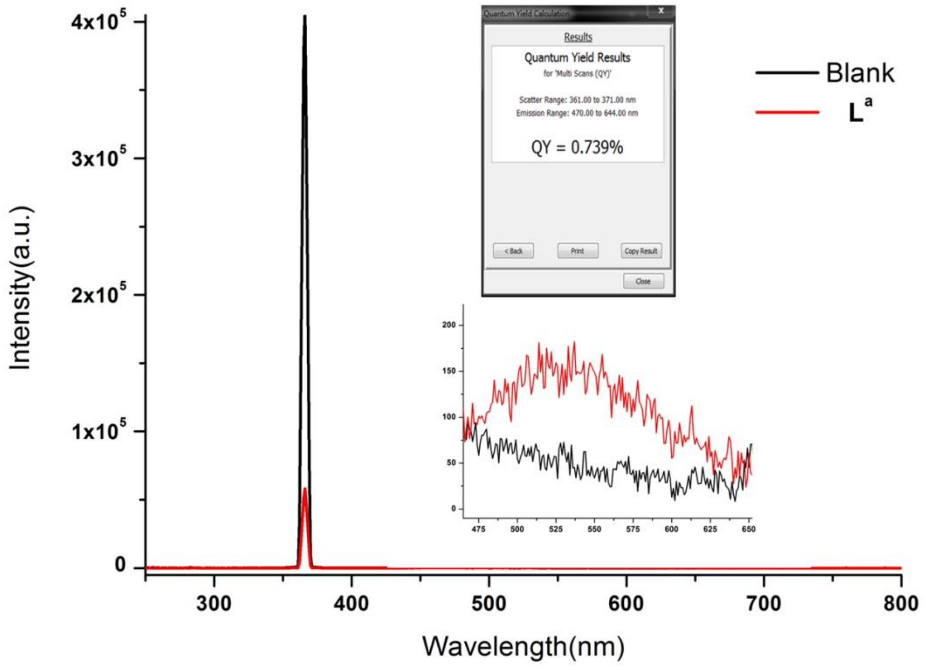

Fig S23. Quantum yield of **L**^{a} in DMSO (298K, λ_{ex}=408nm).

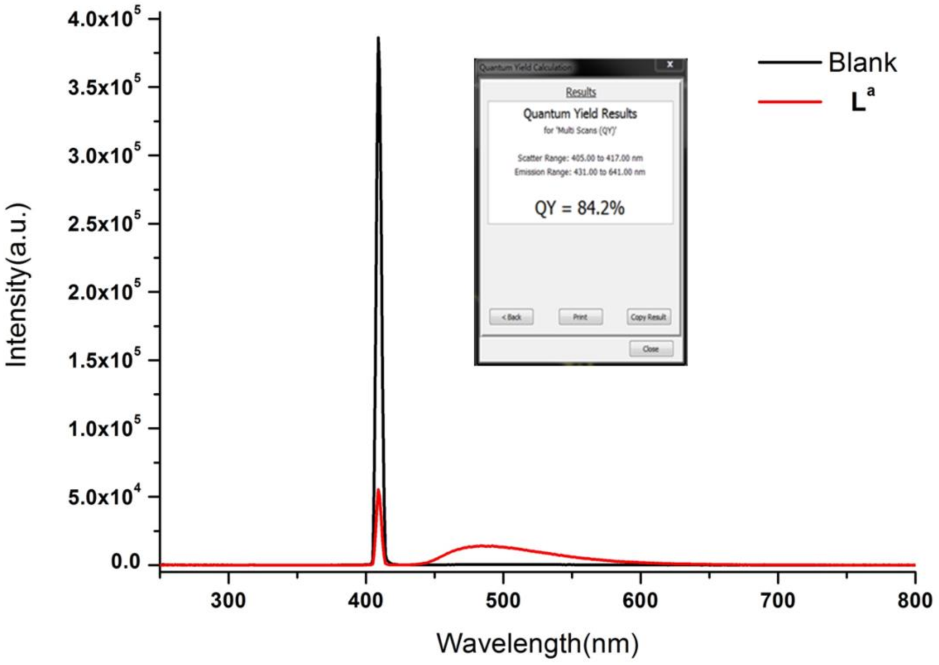

Fig S24. Quantum yield of **L**^{a} in solid state (298K, powder, λ_{ex} = 408 nm).

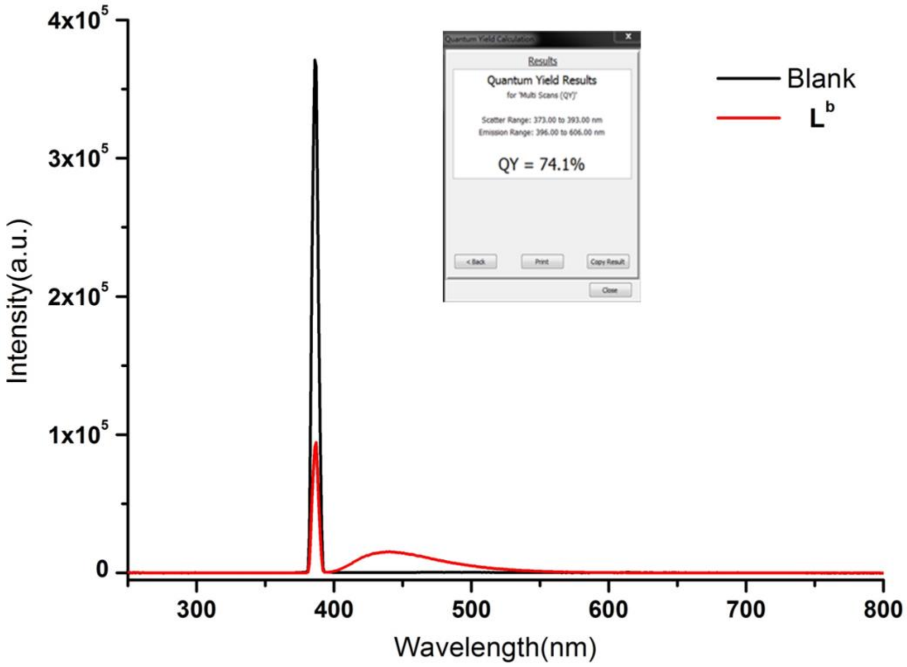

Fig S25. Quantum yield of $\mathbf{L^b}$ in solid state (298K, powder , λ_{ex} = 386 nm).

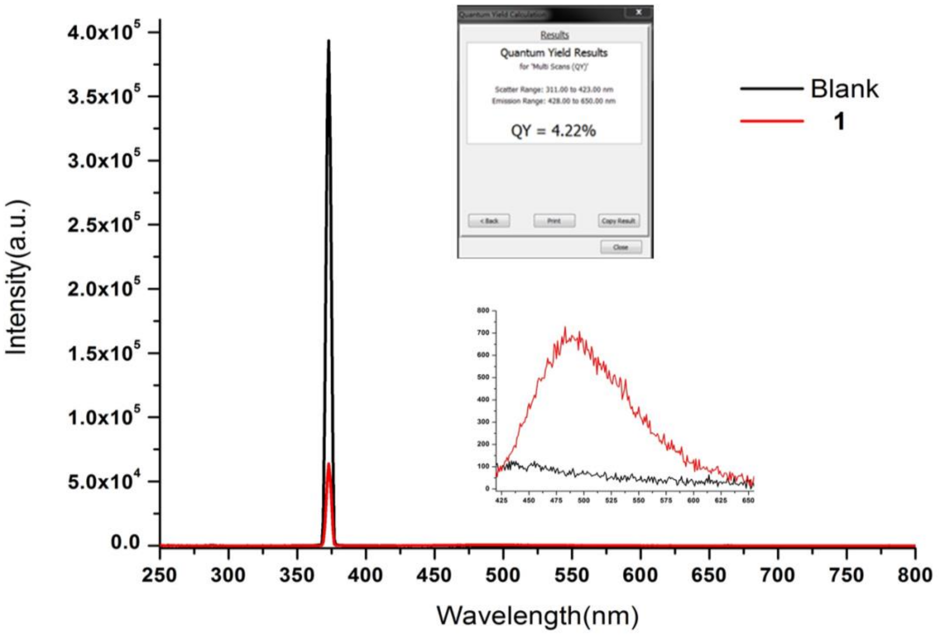

Fig S26. Quantum yield of **1** in H_2O/acetone (v:v=1:1) (298K, λ_{ex}= 372nm).

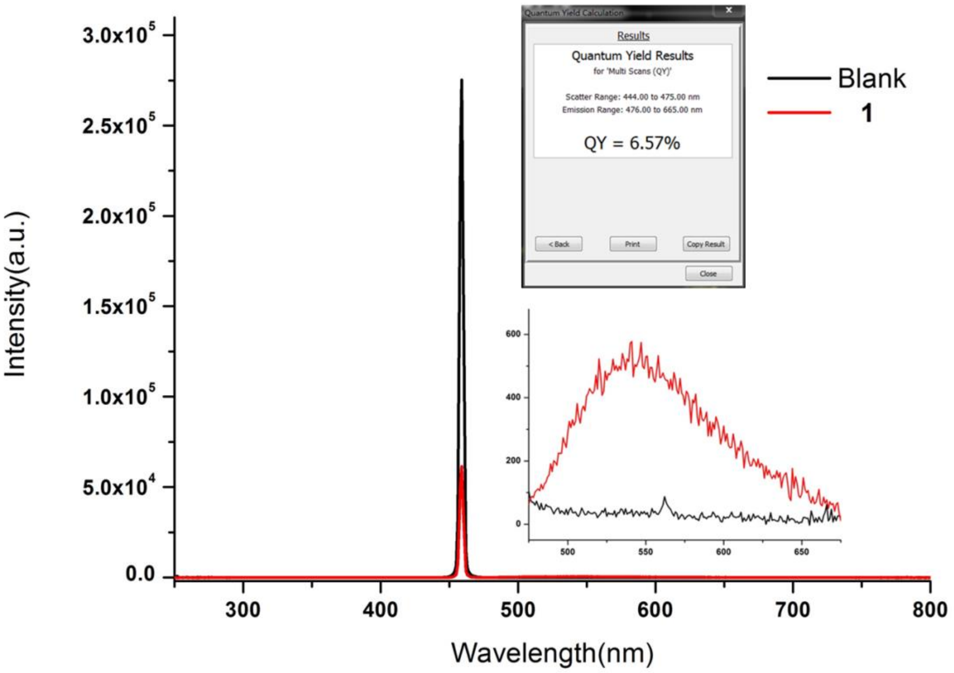

Fig S27. Quantum yield of **1** in solid state (298K, powder, λ_{ex} = 458 nm).

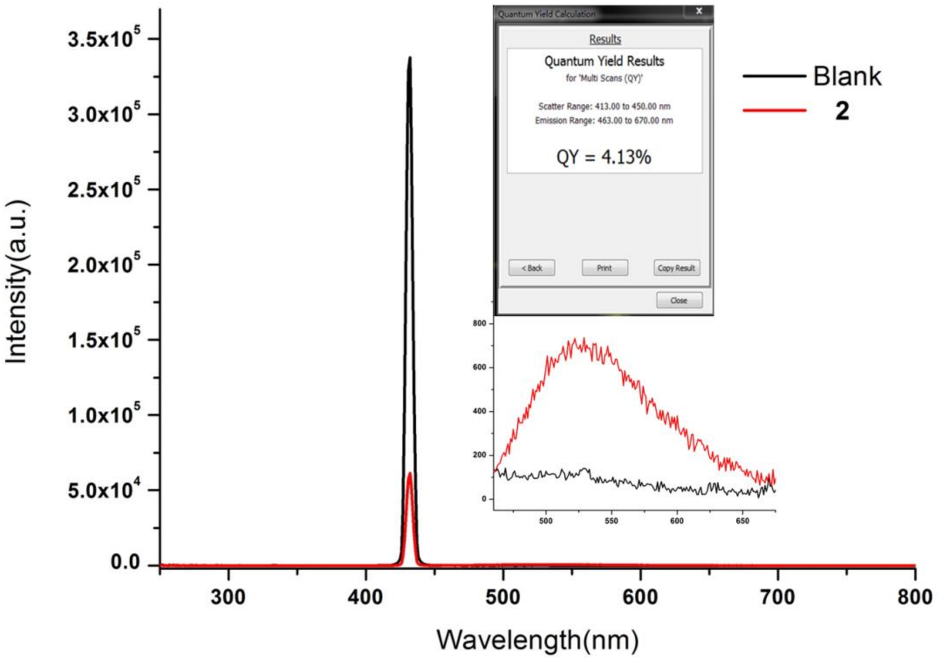

Fig S28. Quantum yield of **2** in solid state (298K, powder, λ_{ex} = 430 nm).
